# Epigenetic age acceleration mediates the association between smoking and diabetes-related outcomes

**DOI:** 10.1186/s13148-023-01512-x

**Published:** 2023-06-03

**Authors:** Xue-Yong Chang, Wan-Yu Lin

**Affiliations:** 1 Institute of Epidemiology and Preventive Medicine, College of Public Health, National Taiwan University, Room 501, No. 17, Xu-Zhou Road, Taipei, 100 Taiwan; 2 Master of Public Health Degree Program, College of Public Health, National Taiwan University, Taipei, Taiwan; 3 Department of Public Health, College of Public Health, National Taiwan University, Taipei, Taiwan

**Keywords:** Aging, Chronological age, DNA methylation, Epigenetic clock, Methylation clock

## Abstract

**Background:**

Smoking can lead to the deterioration of lung function and susceptibility to diabetes. Recently, smoking was found to induce DNA methylation (DNAm) changes in some cytosine-phosphate-guanine sites (CpGs). As linear combinations of DNAm levels of aging-related CpGs, five measures of epigenetic age acceleration (EAA) have received extensive attention: HannumEAA, IEAA, PhenoEAA, GrimEAA, and DunedinPACE. It is of interest to explore whether some measures of EAA can mediate the associations of smoking with diabetes-related outcomes and indices of ventilatory lung function.

**Methods and results:**

In this study, we included self-reported smoking variables (smoking status, the number of pack-years, and years since smoking cessation), seven DNAm markers (HannumEAA, IEAA, PhenoEAA, GrimEAA, DNAm-based smoking pack-years, DNAm plasminogen activator inhibitor 1 [PAI-1] levels, and DunedinPACE), and four health outcomes (fasting glucose, hemoglobin A1C, forced expiratory volume in 1.0 s [FEV1], and forced vital capacity [FVC]) from 2474 Taiwan Biobank participants. Mediation analyses were conducted while adjusting for chronological age, sex, body mass index, drinking status, regular exercise status, educational attainment, and five cell-type proportions. We demonstrated that GrimEAA, DNAm-based smoking pack-years, DNAm PAI-1 levels, DunedinPACE, and PhenoEAA mediated smoking associations with diabetes-related outcomes. Moreover, current and former smoking both had an adverse indirect effect on FVC through DNAm PAI-1 levels. For former smokers, a long time since smoking cessation had a positive indirect impact on FVC through GrimEAA and on FEV1 through PhenoEAA.

**Conclusions:**

This is one of the first studies to comprehensively investigate the role of five measures of EAA in mediating the associations of smoking with the health outcomes of an Asian population. The results showed that the second-generation epigenetic clocks (GrimEAA, DunedinPACE, and PhenoEAA) significantly mediated the associations between smoking and diabetes-related outcomes. In contrast, the first-generation epigenetic clocks (HannumEAA and IEAA) did not significantly mediate any associations of smoking variables with the four health outcomes. Cigarette smoking can, directly and indirectly, deteriorate human health through DNAm changes in aging-related CpG sites.

**Supplementary Information:**

The online version contains supplementary material available at 10.1186/s13148-023-01512-x.

## Introduction

Cigarette smoking can relieve stress and decrease subjective anxiety [1–5]. However, nicotine, the main chemical in cigarettes, affects lung function and leads to chronic obstructive pulmonary disease (COPD) [6, 7]. Furthermore, smoking is also associated with diabetes because of its deleterious effects on pancreatic functions [8–10]. The risks of developing diabetes and COPD are correlated with cigarette consumption [11].

The underlying link between smoking and diseases (such as diabetes [12] and COPD [13]) remains unclear. Smoking leads to two dynamic selection mechanisms [14]. First, smoking increases the risks of diabetes and the deterioration of lung function. Second, smokers with diabetes or worsening lung function are more likely to quit smoking [15]. Therefore, it is necessary to investigate the relationship between cigarettes and glycemic control (or pulmonary function).

As indicated by several epigenome-wide association studies (EWAS) [16–23], smoking is associated with DNA methylation (DNAm) changes at cytosine-phosphate-guanine (CpG) sites across the genome [24, 25]. In the last decade, some CpGs were integrated to predict human biological aging. Five epigenetic clocks have received widespread attention: HannumEAA [26], IEAA [27], PhenoEAA [28], GrimEAA [29, 30], and DunedinPACE [31], where EAA represents “epigenetic age acceleration”.

HannumEAA [26] and IEAA [27] are the so-called “first-generation epigenetic clocks”, as they were developed to predict chronological age. The other three clocks [28–31] are called the “second-generation epigenetic clocks”, which were derived to estimate physiological conditions and aging rates [32]. For example, PhenoEAA [28] is composed of 513 CpGs that can predict phenotypic age, which is a combination of ten indicators for immune, inflammation, metabolic, liver, and kidney conditions. GrimEAA comprises 1,030 CpGs associated with smoking pack-years and seven plasma proteins [29]. Among these plasma proteins, DNAm plasminogen activation inhibitor 1 (PAI-1) (estimated by 211 CpGs) has been shown to outperform GrimEAA in predicting several chronic diseases [29]. Recently, version 2 of GrimEAA [30] additionally included high-sensitivity C-reactive protein and hemoglobin A1C (HbA_1c_) to the original measure [29]. DunedinPACE [31] was derived from 173 CpGs related to declines in organ-system integrity.

GrimEAA and DNAm PAI-1 levels have been reported to be significantly associated with lung function decline (measured by forced expiratory volume in 1.0 s [FEV1] and forced vital capacity [FVC]) [33, 34]. Moreover, DunedinPACE and GrimEAA can reflect diabetes in Taiwanese adults [35]. These findings highlighted that EAA might be a bridge linking smoking to diabetes and lung function reduction.

One mechanism by which active cigarette smoking may influence health is through EAA, a DNAm-based biomarker of aging [36]. Recently, based on a sample of 2978 participants representative of the U.S. population, the three “second-generation epigenetic clocks” were found to mediate a portion of the effects of smoking pack-years on mortality, heart disease, blood pressure levels, and cancer [36]. By analyzing Taiwan Biobank (TWB) DNAm data, we explored whether the five epigenetic clocks can mediate the effects of smoking on fasting glucose (FG), HbA_1c_, FEV1, and FVC. In addition to the five measures of EAA, two GrimAge components were investigated as mediators: DNAm-based smoking pack-years and DNAm PAI-1 levels. The former is more related to smoking, while the latter has been shown to outperform GrimEAA in predicting several age-related traits [29].

## Results

### Basic characteristics

Table 1 shows the primary characteristics of the 2474 TWB participants stratified by smoking status. All the individuals were between 30 and 70 years when participating in the TWB survey. Only 63.7% of the 2474 TWB participants underwent lung function examinations. The measurements of FEV1 and FVC were based on 1576 individuals (1190 non-smokers, 201 former smokers, and 185 current smokers).

**Table 1:** Basic characteristics of the 2474 participants stratified by smoking status

	Non-smokers	Former smokers^a^	Current smokers^b^	*p*-value^c^
Total	1879 (75.95%)	312 (12.61%)	283 (11.44%)	
Age (year)	49.4 (11.2)	52.9 (10.2)	48.8 (10.6)	3.9E-07
Age range	30–70	30–70	30–70	
Sex (male)	736 (39.2%)	272 (87.2%)	235 (83.0%)	3.8E-84
Drinking^d^	72 (3.8%)	30 (9.6%)	69 (24.4%)	1.2E-36
Former smokers’ pack-years	–	12.9 (14.1)	–	
Current smokers’ pack-years	–	–	20.8 (19.9)	
Years since smoking cessation	–	12.9 (10.0)	–	
Regular exercise^e^	819 (43.6%)	178 (57.1%)	95 (33.6%)	3.0E-08
Educational attainment^f^	5.6 (0.9)	5.6 (0.9)	5.4 (0.8)	2.2E-04
BMI (kg/m^2^)	24.1 (3.7)	25.3 (3.2)	25.4 (3.7)	1.3E-14
FG (mg/dL)	94.4 (16.5)	98.9 (20.3)	102.2 (34.0)	8.0E-16
HbA_1c_ (%)	5.7 (0.7)	5.8 (0.7)	5.9 (1.0)	1.7E-04
FEV1 (L)^g^	2.2 (0.8)	2.4 (0.8)	2.5 (0.8)	1.0E-09
FVC (L)^g^	3.0 (0.9)	3.4 (0.7)	3.5 (0.7)	3.9E-28

The characteristics are presented in* n* (%) or mean ± standard deviation

^a^Former smokers were defined as those who “had previously smoked cigarettes for at least 6 months but had quit smoking for at least 6 months when participating in TWB”

^b^Current smokers were defined as those who “had smoked cigarettes for at least 6 months and had not quit smoking when participating in TWB”

^c^The *p*-value of testing the mean or proportion difference among non-smokers, former smokers, and current smokers was based on a Kruskal–Wallis test for continuous variables or a Chi-square test for sex, drinking, and regular exercise

^d^Drinking was defined as those who “had a weekly intake of more than 150 mL of alcoholic beverages for at least 6 months and had not stopped drinking when participating in TWB”

^e^Regular exercise was defined as “performing exercise for 30 min thrice a week”. ‘Exercise’ included leisure-time activities such as jogging, swimming, cycling, yoga, resistance training, hiking, etc.

^f^Educational attainment was an integer ranging from 1 to 7: 1 “illiterate”, 2 “no formal education but literate”, 3 “primary school graduate”, 4 “junior high school graduate”, 5 “senior high school graduate”, 6 “college graduate”, and 7 “Master’s or higher degree”

^g^Only 63.7% of the 2,474 TWB participants underwent lung function examinations. The measurements of FEV1 and FVC were based on 1576 individuals (1190 non-smokers, 201 former smokers, and 185 current smokers)

According to the TWB questionnaire, current smokers were defined as those who “had smoked cigarettes for at least 6 months and had not quit smoking when participating in TWB.” In total, 235 male and 48 female participants were current smokers. Their average number of smoking pack-years was 20.8 (standard deviation [SD] = 19.9).

Former smokers were defined as those who “had previously smoked cigarettes for at least 6 months but had quit smoking for at least 6 months when participating in TWB.” In total, 272 male and 40 female participants were former smokers. Their average number of smoking pack-years was 12.9 (SD = 14.1), and the average time since smoking cessation was 12.9 (SD = 10.0) years.

On average, former smokers (mean age = 52.9, SD = 10.2 years) were older than current smokers (mean age = 48.8, SD = 10.6 years) and non-smokers (mean age = 49.4, SD = 11.2 years) (Kruskal–Wallis test *p-*value = 3.9E-7). There was a strong association between sex and smoking status (Chi-square test *p*-value = 3.8E-84). Over 80% of current and former smokers were males, while more than 60% of non-smokers were females.

Drinking was defined as those who “had a weekly intake of more than 150 mL of alcoholic beverages for at least 6 months and had not stopped drinking when participating in TWB”. The percentage of drinking was largest in current smokers (24.4%) compared with former smokers (9.6%) and non-smokers (3.8%) (Chi-square test *p*-value = 1.2E-36).

Regular exercise was defined as “performing exercise for 30 min thrice a week”. “Exercise” included leisure-time activities such as jogging, swimming, cycling, yoga, resistance training, hiking, etc. The percentage of individuals performing regular exercise was highest in former smokers (57.1%) compared with current smokers (33.6%) and non-smokers (43.6%) (Chi-square test *p*-value = 3.0E-8).

Educational attainment was an integer ranging from 1 to 7: 1 “illiterate”, 2 “no formal education but literate”, 3 “primary school graduate”, 4 “junior high school graduate”, 5 “senior high school graduate”, 6 “college graduate”, and 7 “Master’s or higher degree”. The average educational attainment scores were 5.6 (SD = 0.9), 5.6 (SD = 0.9), and 5.4 (SD = 0.8) for non-smokers, former smokers, and current smokers, respectively. This indicates that the average educational attainment for the 2,474 TWB participants was between “senior high school graduate” and “college graduate”. However, on average, non-smokers and former smokers had higher educational attainment than current smokers (Kruskal–Wallis test *p-*value = 0.00022).

Because of the different male–female proportions across the three groups, current and former smokers demonstrated larger body mass index (BMI) and higher levels of four outcomes (FG, HbA_1c_, FEV1, and FVC) than non-smokers. Given that smoking prevalence and health outcomes significantly vary by sex, we also performed a sensitivity analysis to test the mediation effects in males only (there was insufficient power to implement such tests in females due to their low prevalence of smoking).

### Exposure-mediator relationship (X–> M)

Boxplots of the seven DNAm markers are shown in Figure S1 (Additional file 1). Before performing mediation analysis, we excluded 7, 1, 2, 5, 54, and 1 extreme outliers of HannumEAA, IEAA, PhenoEAA, GrimEAA, DNA-based smoking pack-years, and DunedinPACE, respectively. Extreme outliers were defined by values smaller than ${Q}_{1}-3\times \left({Q}_{3}-{Q}_{1}\right)$ or larger than ${Q}_{3}+3\times \left({Q}_{3}-{Q}_{1}\right)$, where ${Q}_{1}$ and ${Q}_{3}$ are the 25th and 75th percentiles of an EAA, respectively. We detected more extreme outliers in DNA-based smoking pack-years because it demonstrated the most right-skewed distribution among the seven markers (skewness = 1.65, Additional file 1: Figure S1). In contrast, we found no extreme outliers in DNAm PAI-1 levels (skewness = 0.12, Additional file 1: Figure S1).

After removing the extreme outliers, we presented the heatmaps of Pearson’s correlation coefficients among variables in Additional file 1: Figures S2 (both males and females) and S3 (only in males). Because we obtained four measures of EAA (HannumEAA, IEAA, PhenoEAA, and GrimEAA) as the residuals of regressing epigenetic age on chronological age, their correlations with chronological age were close to 0 (Additional file 1: Figure S2). DunedinPACE, DNA-based smoking pack-years, and DNAm PAI-1 levels were positively associated with chronological age (Additional file 1: Figures S2–S3). The analysis focusing on males (Additional file 1: Figure S3) showed that FEV1 and FVC were inversely associated with the second-generation epigenetic clocks (DunedinPACE, GrimEAA, and PhenoEAA) and the two components of GrimEAA (DNA-based smoking pack-years and DNAm PAI-1 levels).

We then evaluated the exposure–mediator relationship (X–> M). Table 2 shows the results of regressing each DNAm marker (M) on smoking variables (X). Table 1 shows that chronological age (in years), sex (male vs. female), BMI (in kg/m^2^), drinking status (yes vs. no), performing regular exercise (yes vs. no), and educational attainment (integer ranging from 1 to 7) are different across the three smoking groups. Therefore, we adjusted for these six covariates in the regression models. Moreover, we also adjusted for five cell-type proportions (B lymphocytes, CD4^+^ T cells, CD8^+^ T cells, monocytes, and natural killer cells) estimated by the Houseman deconvolution method [37]. Cell-type composition is critical because the TWB acquired the DNAm data from peripheral blood rather than other bulk tissues [38]. We had seven DNAm markers and five smoking variables; therefore, *p*-values < 0.05/(7 × 5) = 0.0014 were considered significant.

**Table 2:** Results of regressing each DNAm marker on smoking variables (based on models 1, 3, and 5)

	HannumEAA (in years)		IEAA (in years)		PhenoEAA (in years)		GrimEAA (in years)		DNAm-based smoking pack-years		DNAm PAI-1 (in pg/mL)		DunedinPACE	
	Effect (S.E.)	*p*-value	Effect (S.E.)	*p*-value	Effect (S.E.)	*p*-value	Effect (S.E.)	*p*-value	Effect (S.E.)	*p*-value	Effect (S.E.)	*p*-value	Effect (S.E.)	*p*-value
Former smoking status (yes vs. no) (based on model 1)	0.426 (0.216)	0.0489	0.214 (0.243)	0.3781	**1.011 (0.284)**	**0.0004**	**1.446 (0.187)**	**1.5E-14**	**4.705 (0.338)**	**2.0E-42**	263.389 (131.291)	0.0449	0.019 (0.006)	0.0020
Current smoking status (yes vs. no) (based on model 1)	**1.205 (0.231)**	**2.1E-07**	0.367 (0.260)	0.1582	**1.930 (0.305)**	**2.8E-10**	**5.650 (0.201)**	**1.0E-150**	**14.916 (0.389)**	**9.6E-252**	**1002.309 (140.563)**	**1.3E-12**	**0.084 (0.006)**	**7.9E-38**
Former smokers’ pack-years (based on model 3)	0.021 (0.011)	0.0480	0.021 (0.012)	0.0764	**0.051 (0.014)**	**0.0003**	**0.087 (0.009)**	**5.0E-20**	**0.255 (0.018)**	**1.9E-44**	12.694 (6.549)	0.0527	0.001 (3.0E-04)	0.0017
Current smokers’ pack-years (based on model 3)	**0.027 (0.008)**	**0.0003**	0.003 (0.009)	0.6907	**0.043 (0.010)**	**2.2E-05**	**0.174 (0.007)**	**2.4E-127**	**0.567 (0.018)**	**2.8E-185**	**28.240 (4.629)**	**1.2E-09**	**0.002** **(2.1E-04)**	**5.0E-29**
Years since smoking cessation (based on model 5)	− 0.029 (0.024)	0.2339	− 0.038 (0.027)	0.1555	− 0.091 (0.030)	0.0028	− **0.108 (0.019)**	**2.0E-08**	− **0.313 (0.040)**	**1.2E-13**	− 16.028 (12.937)	0.2164	− 0.001 (0.001)	0.2770

Significant results with *p* < 0.05/(7 × 5) = 0.0014 are highlighted in bold font (7: seven DNAm markers; 5: five smoking variables)

As shown in Table 2, GrimEAA and DNA-based smoking pack-years were associated with all five smoking variables (*p* < 0.0014). PhenoEAA was associated with all smoking variables except years since smoking cessation (*p* = 0.0028). DunedinPACE, DNAm PAI-1 levels, and HannumEAA were positively associated with current smoking status and current smokers’ pack-years. IEAA was not associated with any of the five smoking variables (Table 2). Therefore, IEAA is not a plausible mediator and will be omitted from the following mediation analysis.

Current smokers, on average, were 5.650 years older in GrimEAA (*p* = 1.0E-150), 1.930 years older in PhenoEAA (*p* = 2.8E-10), 1.205 years older in HannumEAA (*p* = 2.1E-07), and had a faster pace of biological aging of 0.084 years per chronological year than non-smokers (*p* = 7.9E-38). Each additional current smokers’ pack-year was associated with 0.174 years larger in GrimEAA (*p* = 2.4E-127), 0.043 years larger in PhenoEAA (*p* = 2.2E-5), 0.027 years larger in HannumEAA (*p* = 0.0003), and a faster pace of biological aging of 0.002 years per chronological year (*p* = 5.0E-29). DNAm-based smoking pack-years and DNAm PAI-1 levels were also significantly associated with current smoking status and current smokers’ pack-years.

Former smoking status and former smokers’ pack-years were associated with DNAm-based smoking pack-years, GrimEAA, and PhenoEAA. Former smokers’ years since smoking cessation were negatively associated with GrimEAA and DNAm-based smoking pack-years. One more year since smoking cessation was associated with a decreased GrimEAA by 0.108 years (*p* = 2.0E-8) and a decreased DNAm-based smoking pack-years by 0.313 (*p* = 1.2E-13).

### Mediation analysis results (X–> M–> Y)

**Fig. 1:**
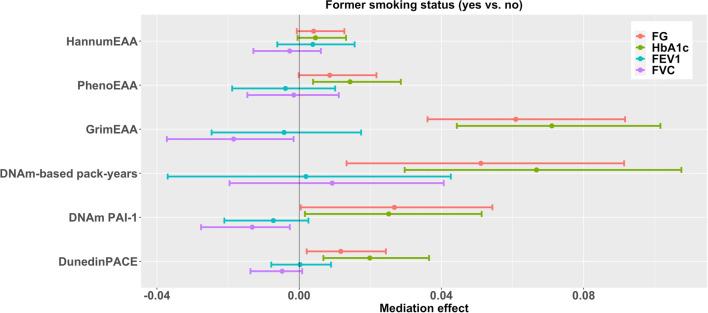
95% Confidence intervals of the effects of six DNAm markers in mediating the associations between former smoking status and four health outcomes. Before mediation analysis, FG and HbA_1c_ were natural-log transformed and then standardized as *z*-scores; FEV1 and FVC were standardized as *z*-scores

**Fig. 2:**
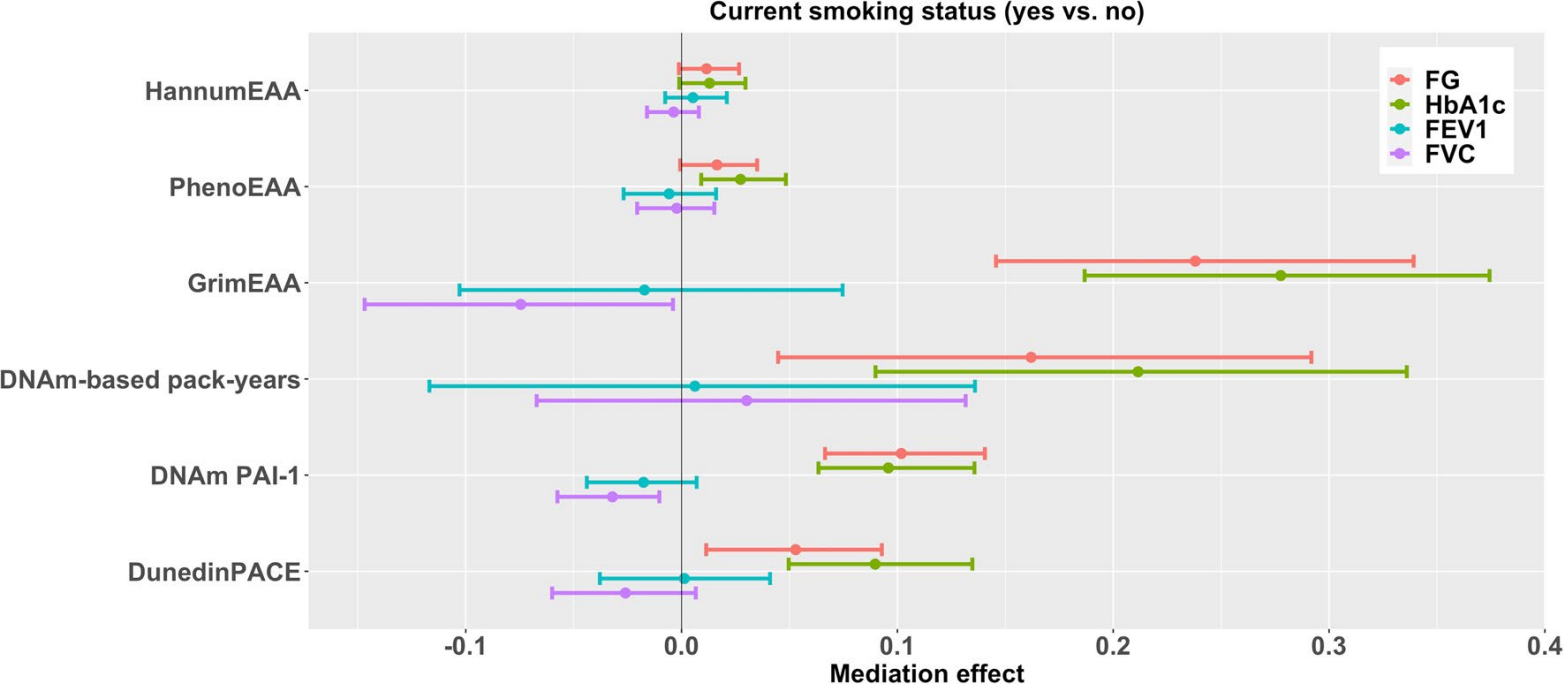
95% Confidence intervals of the effects of six DNAm markers in mediating the associations between current smoking status and four health outcomes. Before mediation analysis, FG and HbA_1c_ were natural-log transformed and then standardized as *z*-scores; FEV1 and FVC were standardized as *z*-scores

**Fig. 3:**
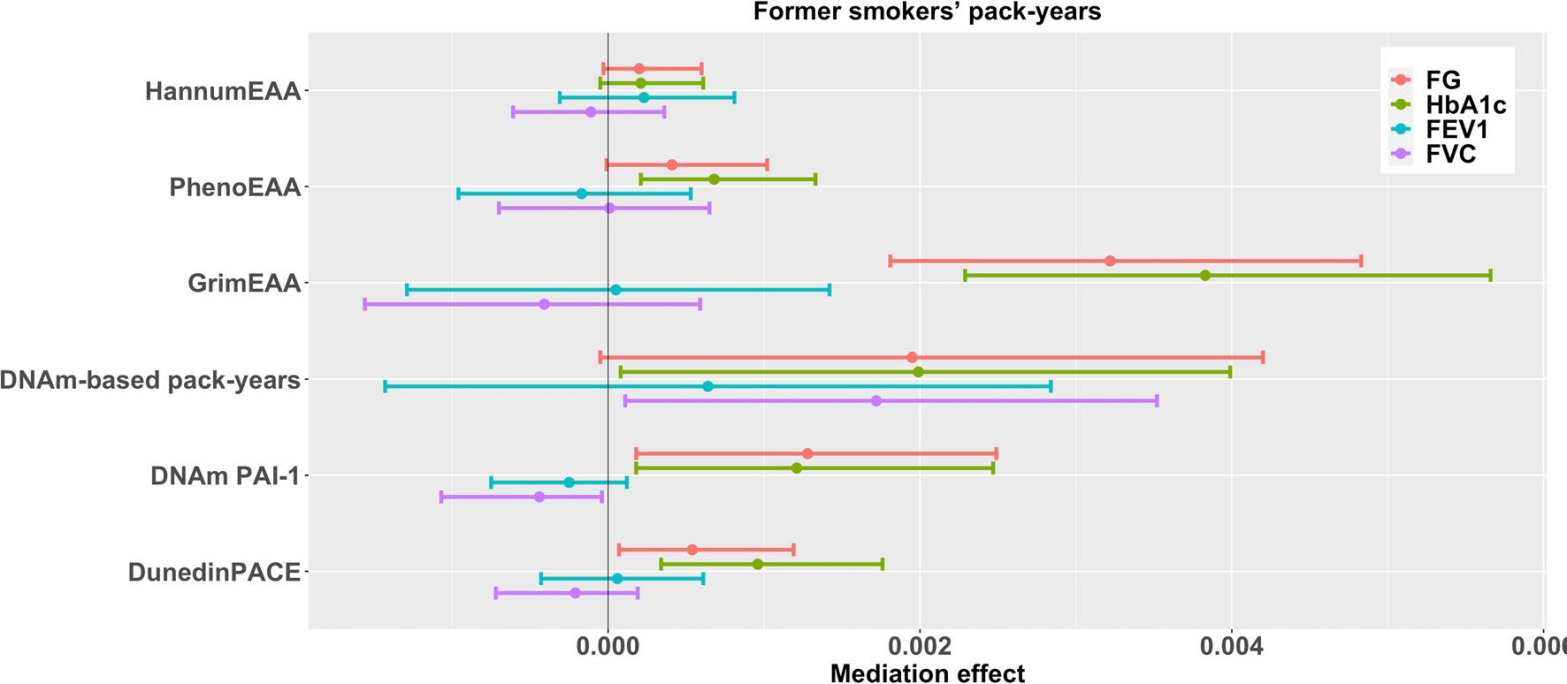
95% Confidence intervals of the effects of six DNAm markers in mediating the associations between former smokers’ pack-years and four health outcomes. Before mediation analysis, FG and HbA_1c_ were natural-log transformed and then standardized as *z*-scores; FEV1 and FVC were standardized as *z*-scores

**Fig. 4:**
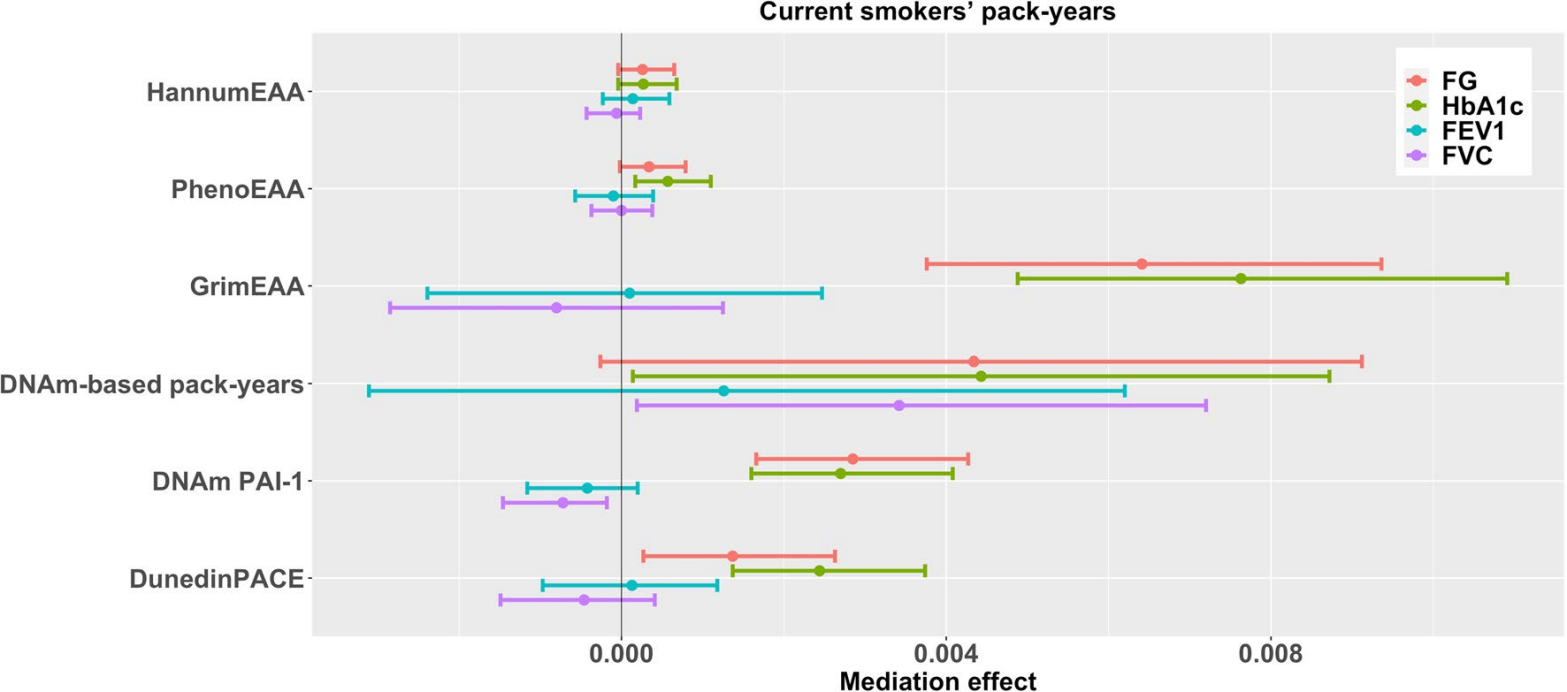
95% Confidence intervals of the effects of six DNAm markers in mediating the associations between current smokers’ pack-years and four health outcomes. Before mediation analysis, FG and HbA_1c_ were natural-log transformed and then standardized as *z*-scores; FEV1 and FVC were standardized as *z*-scores

**Fig. 5:**
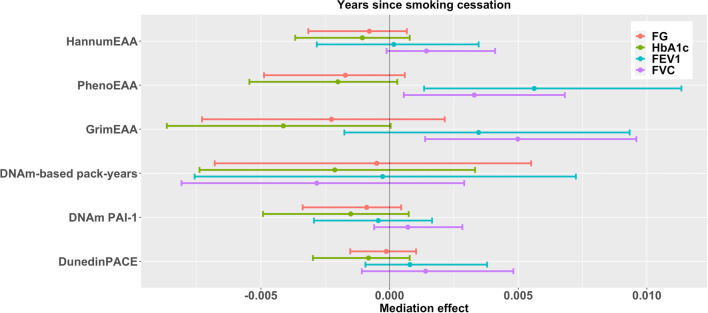
95% Confidence intervals of the effects of six DNAm markers in mediating the associations between years since smoking cessation and four health outcomes. Before mediation analysis, FG and HbA_1c_ were natural-log transformed and then standardized as *z*-scores; FEV1 and FVC were standardized as *z*-scores

Mediation analysis results are shown in Tables 3, 4, 5, 6, 7 and Figs. 1, 2, 3, 4, 5. We first tested the statistical significance of 20 X–Y associations. The total effects of X (smoking variable) on Y (health outcome) were considered significant given the *p*-values < 0.05. We did not adjust for multiple testing in this stage because the total effects of X on Y were not the main objective of this study. Four of the 20 X–Y associations had significant *p*-values of < 0.05: current smoking status on FG (*p* = 0.00012, Table 4) and HbA_1c_ (*p* = 0.048, Table 4); current smokers’ pack-years on FG (*p* = 1.4E-7, Table 6) and HbA_1c_ (*p* = 0.003, Table 6).

**Table 3:** Results of six DNAm markers in mediating the associations between former smoking status and four health outcomes

Outcome^a^	Total effect	95% Confidence interval		*p*-value	Sample size
FG	0.0442	− 0.0761	0.1644	0.471	2469
HbA_1c_	0.0253	− 0.0941	0.1448	0.677	2469
FEV1	− 0.0820	− 0.2155	0.0516	0.229	1576
FVC	0.0427	− 0.0667	0.1520	0.444	1576

Outcome	Mediator	Mediation effect^b^	95% confidence interval		FDR^c^	Proportion mediated^d^ (%)	Sample size
FG	HannumEAA	0.0041	− 0.0007	0.0127	0.264	9.3	2462
HbA_1c_		0.0046	− 0.0005	0.0132	0.209	18.2	2462
FEV1		0.0038	− 0.0062	0.0156	0.597	− 4.6	1569
FVC		− 0.0027	− 0.0129	0.0061	0.710	− 6.3	1569
FG	PhenoEAA	0.0086	− 0.0001	0.0217	0.138	19.5	2467
HbA_1c_		**0.0143**	**0.0039**	**0.0286**	**0.021**	56.5	2467
FEV1		− 0.0039	− 0.0188	0.0101	0.710	4.8	1574
FVC		− 0.0015	− 0.0146	0.0111	0.927	− 3.5	1574
FG	GrimEAA	**0.0609**	**0.0361**	**0.0916**	** < 0.001**	137.8	2464
HbA_1c_		**0.0710**	**0.0443**	**0.1016**	** < 0.001**	280.6	2464
FEV1		− 0.0043	− 0.0246	0.0174	0.816	5.2	1572
FVC		− 0.0184	− 0.0372	− 0.0016	0.108	− 43.1	1572
FG	DNAm-based pack-years	**0.0511**	**0.0133**	**0.0914**	**0.028**	115.6	2415
HbA_1c_		**0.0667**	**0.0297**	**0.1075**	** < 0.001**	263.6	2415
FEV1		0.0019	− 0.0370	0.0427	0.957	− 2.3	1538
FVC		0.0093	− 0.0196	0.0406	0.707	21.8	1538
FG	DNAm PAI-1	0.0268	0.0005	0.0543	0.120	60.6	2469
HbA_1c_		0.0252	0.0016	0.0513	0.117	99.6	2469
FEV1		− 0.0073	− 0.0210	0.0026	0.294	8.9	1576
FVC		− **0.0132**	− **0.0276**	− **0.0026**	**0.026**	− 30.9	1576
FG	DunedinPACE	**0.0117**	**0.0021**	**0.0244**	**0.024**	26.5	2468
HbA_1c_		**0.0199**	**0.0068**	**0.0365**	**0.007**	78.7	2468
FEV1		0.0002	− 0.0079	0.0090	0.957	− 0.2	1575
FVC		− 0.0048	− 0.0137	0.0009	0.246	− 11.2	1575

^a^Before mediation analysis, FG and HbA_1c_ were natural-log transformed and then standardized as *z*-scores; FEV1 and FVC were standardized as *z*-scores

^b^Mediation effect is the effect of X (former smoking status) on Y (health outcome) through the mediator (DNAm marker)

^c^FDR (false discovery rate) represents the FDR-adjusted *p*-value. Significant effects with FDR < 0.05 are marked in bold

^d^Proportion mediated was calculated by dividing the mediation effect by the total effect

**Table 4:** Results of six DNAm markers in mediating the associations between current smoking status and four health outcomes

Outcome ^a^	Total effect	95% confidence interval		*p*-value	Sample size
FG	**0.2533**	**0.1245**	**0.3820**	**1.2E-04**	2469
HbA_1c_	**0.1293**	**0.0014**	**0.2572**	**0.048**	2469
FEV1	0.0208	− 0.1229	0.1645	0.777	1576
FVC	0.0826	− 0.0350	0.2003	0.169	1576

Outcome	Mediator	Mediation effect^b^	95% confidence interval		FDR^c^	Proportion mediated^d^ (%)	Sample size
FG	HannumEAA	0.0116	− 0.0013	0.0267	0.183	4.6	2462
HbA_1c_		0.0130	− 0.0011	0.0296	0.145	10.1	2462
FEV1		0.0053	− 0.0076	0.0210	0.590	25.5	1569
FVC		− 0.0037	− 0.0160	0.0080	0.707	− 4.5	1569
FG	PhenoEAA	0.0164	− 0.0007	0.0350	0.145	6.5	2467
HbA_1c_		**0.0273**	**0.0091**	**0.0483**	**0.013**	21.1	2467
FEV1		− 0.0058	− 0.0269	0.0160	0.767	− 27.9	1574
FVC		− 0.0023	− 0.0206	0.0152	0.927	− 2.8	1574
FG	GrimEAA	**0.2381**	**0.1457**	**0.3392**	** < 0.001**	94.0	2464
HbA_1c_		**0.2776**	**0.1868**	**0.3744**	** < 0.001**	214.7	2464
FEV1		− 0.0172	− 0.1029	0.0746	0.840	− 82.7	1572
FVC		− 0.0745	− 0.1468	− 0.0040	0.108	− 90.2	1572
FG	DNAm-based pack-years	**0.1620**	**0.0448**	**0.2918**	**0.028**	64.0	2415
HbA_1c_		**0.2115**	**0.0899**	**0.3360**	**0.007**	163.6	2415
FEV1		0.0062	− 0.1168	0.1360	0.957	29.8	1538
FVC		0.0302	− 0.0671	0.1316	0.710	36.6	1538
FG	DNAm PAI-1	**0.1018**	**0.0665**	**0.1406**	** < 0.001**	40.2	2469
HbA_1c_		**0.0958**	**0.0634**	**0.1357**	** < 0.001**	74.1	2469
FEV1		− 0.0176	− 0.0439	0.0070	0.285	− 84.6	1576
FVC		− **0.0320**	− **0.0575**	− **0.0103**	**0.016**	− 38.7	1576
FG	DunedinPACE	**0.0529**	**0.0114**	**0.0928**	**0.045**	20.9	2468
HbA_1c_		**0.0897**	**0.0496**	**0.1347**	** < 0.001**	69.4	2468
FEV1		0.0013	− 0.0379	0.0410	0.957	6.3	1575
FVC		− 0.0260	− 0.0600	0.0066	0.254	− 31.5	1575

^a^Before mediation analysis, FG and HbA_1c_ were natural-log transformed and then standardized as *z*-scores; FEV1 and FVC were standardized as *z*-scores

^b^Mediation effect is the effect of X (current smoking status) on Y (health outcome) through the mediator (DNAm marker)

^c^FDR (false discovery rate) represents the FDR-adjusted *p*-value. Significant effects with FDR < 0.05 are marked in bold

^d^Proportion mediated was calculated by dividing the mediation effect by the total effect

The significance of the X–Y association is not a requirement for the following X–> M–> Y mediation analysis [39, 40] because (1) when the effect size is small, the sample size may not be sufficient to detect the effect of X on Y [40]; and (2) two or more indirect effects with opposite directions may cancel each other out, producing an insignificant total effect [41].

Former smoking status, former smokers’ pack-years, and years since smoking cessation were not significantly associated with health outcomes (Tables 3, 5, and 7). However, our analysis of all 173,807 TWB participants (Additional file 1: Table S1) showed that 18 of the 20 X–Y associations were significant (*p* < 0.05; Additional file 1: Table S2). Moreover, according to previous studies, the time since smoking cessation is inversely associated with diabetes risk [42]; former smoking is related to lasting damage to lung functions [43]. Our analysis of all 173,807 TWB participants and these prior findings encouraged us to continue the mediation analysis even when the X–Y associations were insignificant in the sample of 2474 individuals.

**Table 5:** Results of six DNAm markers in mediating the associations between former smokers’ pack-years and four health outcomes

Outcome^a^	Total effect	95% confidence interval		*p*-value	Sample size
FG	0.0046	− 0.0013	0.0106	0.129	2452
HbA_1c_	0.0050	− 0.0010	0.0109	0.102	2452
FEV1	− 0.0017	− 0.0084	0.0051	0.629	1563
FVC	− 0.0010	− 0.0065	0.0045	0.726	1563

Outcome	Mediator	Mediation effect^b^	95% confidence interval		FDR^c^	Proportion mediated^d^ (%)	Sample size
FG	HannumEAA	0.0002	− 3.0E-05	0.0006	0.264	4.3	2445
HbA_1c_		0.0002	− 0.0001	0.0006	0.259	4.0	2445
FEV1		0.0002	− 0.0003	0.0008	0.575	− 11.8	1556
FVC		− 0.0001	− 0.0006	0.0004	0.767	10.0	1556
FG	PhenoEAA	0.0004	− 1.0E-05	0.0010	0.138	8.7	2450
HbA_1c_		**0.0007**	**0.0002**	**0.0013**	**0.013**	14.0	2450
FEV1		− 0.0002	− 0.0010	0.0005	0.767	11.8	1561
FVC		1.0E-05	− 0.0007	0.0007	0.968	− 1.0	1561
FG	GrimEAA	**0.0032**	**0.0018**	**0.0048**	** < 0.001**	69.6	2448
HbA_1c_		**0.0038**	**0.0023**	**0.0057**	** < 0.001**	76.0	2448
FEV1		0.0001	− 0.0013	0.0014	0.957	− 5.9	1560
FVC		− 0.0004	− 0.0016	0.0006	0.590	40.0	1560
FG	DNAm-based pack-years	0.0020	− 0.0001	0.0042	0.145	43.5	2399
HbA_1c_		0.0020	0.0001	0.0040	0.117	40.0	2399
FEV1		0.0006	− 0.0014	0.0028	0.710	− 35.3	1526
FVC		0.0017	0.0001	0.0035	0.108	− 170.0	1526
FG	DNAm PAI-1	0.0013	0.0002	0.0025	0.062	28.3	2452
HbA_1c_		0.0012	0.0002	0.0025	0.070	24.0	2452
FEV1		− 0.0003	− 0.0008	0.0001	0.336	17.6	1563
FVC		− 0.0004	− 0.0011	− 4.0E-05	0.101	40.0	1563
FG	DunedinPACE	0.0005	0.0001	0.0012	0.062	10.9	2451
HbA_1c_		**0.0010**	**0.0003**	**0.0018**	**0.016**	20.0	2451
FEV1		0.0001	− 0.0004	0.0006	0.906	− 5.9	1562
FVC		− 0.0002	− 0.0007	0.0002	0.501	20.0	1562

^a^Before mediation analysis, FG and HbA_1c_ were natural-log transformed and then standardized as *z*-scores; FEV1 and FVC were standardized as *z*-scores

^b^Mediation effect is the effect of X (former smokers’ pack-years) on Y (health outcome) through the mediator (DNAm marker)

^c^FDR (false discovery rate) represents the FDR-adjusted *p*-value. Significant effects with FDR < 0.05 are marked in bold

^d^Proportion mediated was calculated by dividing the mediation effect by the total effect

No significant mediation effects were observed in HannumEAA (false discovery rate [FDR] > 0.05, Tables 3, 4, 5, 6, 7, Figs. 1, 2, 3, 4, 5). The associations between current smoking and diabetes-related outcomes (FG and HbA_1c_) were significantly mediated by GrimEAA, DNAm-based smoking pack-years, DNAm PAI-1 levels, DunedinPACE, and PhenoEAA (only HbA_1c_) (FDR < 0.05, Table 4 and Fig. 2). GrimEAA mediated 94.0% of current smoking’s effect on FG and 214.7% of current smoking’s effect on HbA_1c_. DNAm-based smoking pack-years mediated 64.0% of current smoking’s impact on FG and 163.6% of current smoking’s impact on HbA_1c_. DNAm PAI-1 levels mediated 40.2% of current smoking’s effect on FG and 74.1% of current smoking’s effect on HbA_1c_. DunedinPACE mediated 20.9% of current smoking’s impact on FG and 69.4% of current smoking’s impact on HbA_1c_. PhenoEAA mediated 21.1% of current smoking’s effect on HbA_1c_.

The proportion mediated was over 100% because the direct and mediation effects were in opposite directions. Taking current smoking status – > GrimEAA –> HbA_1c_ as an example, the mediation effect was 0.2776 (Table 4), the direct effect was − 0.1483 (*p* = 0.083), and the total effect was 0.2776–0.1483 = 0.1293 (Table 4).

The associations between current smokers’ pack-years and diabetes-related outcomes (FG and HbA_1c_) were significantly mediated by GrimEAA, DNAm PAI-1 levels, DunedinPACE, and PhenoEAA (only HbA_1c_) (FDR < 0.05, Table 6 and Fig. 4). GrimEAA mediated 56.1% of the total effect of current smokers’ pack-years on FG and 120.6% of the total effect of current smokers’ pack-years on HbA_1c_. DNAm PAI-1 mediated 25.4% of the total impact of current smokers’ pack-years on FG and 42.9% of the total impact of current smokers’ pack-years on HbA_1c_. DunedinPACE mediated 12.3% of the total effect of current smokers’ pack-years on FG and 38.1% of the total effect of current smokers’ pack-years on HbA_1c_. PhenoEAA mediated 9.5% of the total impact of current smokers’ pack-years on HbA_1c_.

**Table 6:** Results of six DNAm markers in mediating the associations between current smokers’ pack-years and four health outcomes

Outcome ^a^	Total effect	95% confidence interval		*p*-value	Sample size
FG	**0.0114**	**0.0072**	**0.0156**	**1.4E-07**	2452
HbA_1c_	**0.0063**	**0.0021**	**0.0105**	**0.003**	2452
FEV1	− 0.0006	− 0.0052	0.0039	0.782	1563
FVC	− 0.0011	− 0.0048	0.0026	0.565	1563

Outcome	Mediator	Mediation effect^b^	95% confidence interval		FDR^c^	Proportion mediated^d^	Sample size
FG	HannumEAA	0.0003	− 4.0E-05	0.0007	0.194	2.6	2445
HbA_1c_		0.0003	− 4.0E-05	0.0007	0.178	4.8	2445
FEV1		0.0001	− 0.0002	0.0006	0.599	− 16.7	1556
FVC		− 0.0001	− 0.0004	0.0002	0.767	9.1	1556
FG	PhenoEAA	0.0003	− 2.0E-05	0.0008	0.145	2.6	2450
HbA_1c_		**0.0006**	**0.0002**	**0.0011**	**0.016**	9.5	2450
FEV1		− 0.0001	− 0.0006	0.0004	0.767	16.7	1561
FVC		0.0000	− 0.0004	0.0004	0.957	0.0	1561
FG	GrimEAA	**0.0064**	**0.0038**	**0.0094**	** < 0.001**	56.1	2448
HbA_1c_		**0.0076**	**0.0049**	**0.0109**	** < 0.001**	120.6	2448
FEV1		0.0001	− 0.0024	0.0025	0.957	− 16.7	1560
FVC		− 0.0008	− 0.0029	0.0013	0.622	72.7	1560
FG	DNAm-based pack-years	0.0043	− 0.0003	0.0091	0.145	37.7	2399
HbA_1c_		0.0044	0.0001	0.0087	0.120	69.8	2399
FEV1		0.0013	− 0.0031	0.0062	0.744	− 216.7	1526
FVC		0.0034	0.0002	0.0072	0.108	− 309.1	1526
FG	DNAm PAI-1	**0.0029**	**0.0017**	**0.0043**	** < 0.001**	25.4	2452
HbA_1c_		**0.0027**	**0.0016**	**0.0041**	** < 0.001**	42.9	2452
FEV1		− 0.0004	− 0.0012	0.0002	0.332	66.7	1563
FVC		− **0.0007**	− **0.0015**	− **0.0002**	**0.024**	63.6	1563
FG	DunedinPACE	**0.0014**	**0.0003**	**0.0026**	**0.045**	12.3	2451
HbA_1c_		**0.0024**	**0.0014**	**0.0037**	** < 0.001**	38.1	2451
FEV1		0.0001	− 0.0010	0.0012	0.927	− 16.7	1562
FVC		− 0.0005	− 0.0015	0.0004	0.501	45.5	1562

^a^Before mediation analysis, FG and HbA_1c_ were natural-log transformed and then standardized as *z*-scores; FEV1 and FVC were standardized as *z*-scores

^b^Mediation effect is the effect of X (current smokers’ pack-years) on Y (health outcome) through the mediator (DNAm marker)

^c^FDR (false discovery rate) represents the FDR-adjusted *p*-value. Significant effects with FDR < 0.05 are marked in bold

^d^Proportion mediated was calculated by dividing the mediation effect by the total effect

**Table 7:** Results of six DNAm markers in mediating the associations between years since smoking cessation and four health outcomes

Outcome ^a^	Total effect	95% confidence interval		*p*-value	Sample size
FG	0.0012	− 0.0123	0.0147	0.859	300
HbA_1c_	− 0.0018	− 0.0150	0.0115	0.795	300
FEV1	0.0005	− 0.0143	0.0154	0.947	193
FVC	− 0.0036	− 0.0147	0.0074	0.516	193

Outcome	Mediator	Mediation effect^b^	95% confidence interval		FDR^c^	Proportion mediated^d^ (%)	Sample size
FG	HannumEAA	− 0.0008	− 0.0032	0.0007	0.566	− 66.7	299
HbA_1c_		− 0.0011	− 0.0037	0.0008	0.447	61.1	299
FEV1		0.0002	− 0.0028	0.0035	0.957	40.0	192
FVC		0.0014	− 0.0001	0.0041	0.191	− 38.9	192
FG	PhenoEAA	− 0.0017	− 0.0049	0.0006	0.273	− 141.7	300
HbA_1c_		− 0.0020	− 0.0055	0.0003	0.194	111.1	300
FEV1		**0.0056**	**0.0013**	**0.0113**	**0.026**	1120.0	193
FVC		0.0033	0.0006	0.0068	0.055	− 91.7	193
FG	GrimEAA	− 0.0023	− 0.0073	0.0021	0.501	− 191.7	300
HbA_1c_		− 0.0041	− 0.0087	4.0E-05	0.138	227.8	300
FEV1		0.0035	− 0.0018	0.0093	0.336	700.0	193
FVC		**0.0050**	**0.0014**	**0.0096**	**0.026**	− 138.9	193
FG	DNAm-based pack-years	− 0.0005	− 0.0068	0.0055	0.934	− 41.7	298
HbA_1c_		− 0.0021	− 0.0074	0.0033	0.599	116.7	298
FEV1		− 0.0003	− 0.0076	0.0072	0.957	− 60.0	192
FVC		− 0.0028	− 0.0081	0.0029	0.501	77.8	192
FG	DNAm PAI-1	− 0.0009	− 0.0034	0.0005	0.443	− 75.0	300
HbA_1c_		− 0.0015	− 0.0049	0.0007	0.345	83.3	300
FEV1		− 0.0004	− 0.0029	0.0017	0.767	− 80.0	193
FVC		0.0007	− 0.0006	0.0028	0.512	− 19.4	193
FG	DunedinPACE	− 0.0001	− 0.0015	0.0010	0.934	− 8.3	300
HbA_1c_		− 0.0008	− 0.0030	0.0008	0.501	44.4	300
FEV1		0.0008	− 0.0009	0.0038	0.599	160.0	193
FVC		0.0014	− 0.0011	0.0048	0.467	− 38.9	193

^a^Before mediation analysis, FG and HbA_1c_ were natural-log transformed and then standardized as *z*-scores; FEV1 and FVC were standardized as *z*-scores

^b^Mediation effect is the effect of X (years since smoking cessation) on Y (health outcome) through the mediator (EAA)

^c^FDR (false discovery rate) represents the FDR-adjusted *p*-value. Significant effects with FDR < 0.05 are marked in bold

^d^Proportion mediated was calculated by dividing the mediation effect by the total effect

Moreover, years since smoking cessation positively affected FVC through GrimEAA (FDR = 0.026) and FEV1 through PhenoEAA (FDR = 0.026, Table 7 and Fig. 5). Former smoking had an adverse indirect effect on diabetes-related outcomes through GrimEAA, DNAm-based smoking pack-years, DunedinPACE, and PhenoEAA (only HbA_1c_), and former smoking exerted an indirect negative impact on FVC through DNAm PAI-1 levels (FDR < 0.05, Table 3 and Fig. 1). Longer former smokers’ pack-years had an adverse indirect effect on diabetes-related outcomes through GrimEAA, DunedinPACE (only HbA_1c_), and PhenoEAA (only HbA_1c_) (FDR < 0.05, Table 5 and Fig. 3).

Among the 2474 individuals, 1243 were males. The primary characteristics of the 1243 male participants stratified by smoking status are shown in Additional file 1: Table S3. The exposure-mediator relationship (X–> M, Additional file 1: Table S4) and the mediation analysis results for male participants (X–> M–> Y, Additional file 1: Tables S5–S9 and Figures S4–S8) are similar to those obtained from both males and females (Tables 2, 3, 4, 5, 6, 7 and Figs. 1, 2, 3, 4, 5).

## Discussion

Previous research suggests that DNAm is a plausible biological pathway linking smoking exposure to health span [36]. By analyzing the DNAm data of 2978 participants from the Health and Retirement Study (HRS) 2016 Venous Blood Study, a recent study showed that the three second-generation epigenetic clocks (PhenoEAA, GrimEAA, and DunedinPoAm38 [the previous version of DunedinPACE]) [28, 29, 31, 44] mediated the effects of smoking pack-years on cancer, heart disease, high blood pressure, and mortality [36]. Moreover, GrimEAA and DunedinPoAm38 also mediated the association between smoking and lung diseases such as emphysema or chronic bronchitis [36]. The first-generation epigenetic clocks (HannumEAA and IEAA) [26, 27] were not mediators of the association between smoking exposure and any health outcomes in that study (cancer, heart disease, high blood pressure, lung disease, and mortality) [36]. These results were based on 2,978 U.S. adults over 50 years old (mean age = 67.65; SD = 9.61 years; range: 50–100). 73% of the sample were non-Hispanic White, 12% were non-Hispanic Black, 11% were Hispanic, and 4% were other non-Hispanic ethnicities.

In our study, by analyzing the DNAm data of 2474 TWB participants, we showed that the associations between current smoking and diabetes-related indicators (FG and HbA_1c_) were mediated by GrimEAA, DunedinPACE, and PhenoEAA (Tables 4 and 6, Figs. 2 and 4). A plausible mediator should meet two requirements: (1) the association between smoking (X) and EAA (M); and (2) the association between EAA (M) and the health outcome (Y, here, diabetes-related indicators) while controlling for smoking status (X). GrimEAA was derived from 1030 CpGs associated with smoking pack-years and nine plasma proteins [30]. GrimEAA and its component (DNAm-based smoking pack-years) are the most related to the five smoking variables (Table 2). They are good mediators of the association between smoking and diabetes-related indicators. To our knowledge, this is the first study linking smoking with diabetes through EAA.

We also show that former smoking adversely affects diabetes-related outcomes through the second-generation epigenetic clocks and FVC through DNAm PAI-1 levels (Table 3). However, quitting smoking earlier positively affects FVC through GrimEAA and FEV1 through PhenoEAA (Table 7). Nonetheless, this is a cross-sectional study, and no temporal relationship exists between EAA and health outcomes. We cannot exclude the possibility that adverse health outcomes (i.e., diabetes or reduced ventilatory lung function) lead to altered EAA.

Although we here evaluate different outcomes, our results are consistent with previous findings [36] that the second-generation epigenetic clocks are mediators of the association between smoking exposure and health outcomes (cancer, heart disease, high blood pressure, lung disease, and mortality in [36]; FG, HbA_1c_, FEV1, and FVC in the present study). European data have suggested that GrimEAA and DNAm PAI-1 levels are inversely associated with FVC [33]. This result aligns with our finding that smoking-related variables indirectly affect FVC through GrimEAA (Table 7) and DNAm PAI-1 levels (Tables 3, 4, and 6).

Biomarkers such as EAA can help figure out the mechanism linking smoking to health outcomes. Different from the first-generation epigenetic clocks (HannumEAA and IEAA) [26, 27], the second-generation epigenetic clocks (PhenoEAA, GrimEAA, and DunedinPACE) were derived from two-stage approaches. The first stage was to build some physiological measures, and the following step was to select CpGs that can predict the physiological measures [28–31]. Therefore, compared with the first-generation epigenetic clocks, the second-generation clocks were more associated with health outcomes while controlling for the smoking variables, meeting the second requirement of a mediator.

In this work, GrimEAA indicated the EAA based on version 2 of DNAm GrimAge [30]. In the 20 mediation analyses (X–> GrimEAA–> Y, where X and Y are five smoking variables and four health outcomes, respectively), version 2 of GrimEAA [30] exhibited improvements over version 1 of GrimEAA [29]. Specifically, version 2 of GrimEAA showed eleven lower mediation *p*-values, two larger mediation *p*-values, and seven identical *p*-values < 0.001 (Additional file 1: Figure S9) compared with version 1 of GrimEAA. We also observed similar results when the analysis focused on males (Additional file 1: Figure S10). Version 2 of GrimEAA is a better mediator than version 1 of GrimEAA regarding the research topic in this study.

This is one of the first studies to show that the second-generation epigenetic clocks can mediate smoking associations with diabetes-related outcomes, based on rarely reported Asian data. Compared with other studies treating EAA as a mediator [36, 45], our study was unique in analyzing an East Asian sample. Moreover, our study sample was relatively younger (mean age = 49.76; SD = 11.08 years; range: 30–70) than that derived from the HRS 2016 Venous Blood Study [36, 45]. Finally, we investigated different health outcomes from previous studies [36, 45]. This work can fill the research gap regarding the role of EAA as a mediator between smoking and health.

## Conclusions

In this study, we comprehensively investigated the ability of the five measures of EAA to mediate the associations of smoking with four health outcomes of an East Asian population. The results showed that the second-generation epigenetic clocks (GrimEAA, DunedinPACE, and PhenoEAA) significantly mediated the associations between smoking and diabetes-related outcomes. Former smoking adversely affected diabetes-related outcomes through the second-generation epigenetic clocks and FVC through DNAm PAI-1 levels. However, quitting smoking earlier positively affected FVC through GrimEAA and FEV1 through PhenoEAA. In contrast, the first-generation epigenetic clocks (HannumEAA and IEAA) did not significantly mediate any effects of smoking variables on the four health outcomes (FG, HbA_1c_, FEV1, and FVC). Active cigarette smoking can, directly and indirectly, deteriorate human health through DNAm changes in aging-related CpG sites.

## Methods

### Study participants

Since October 2012, the TWB has recruited 173,807 community-based volunteers living in Taiwan. According to Taiwan’s male–female proportion and the population size in each county, 2474 individuals were randomly selected from all TWB participants to undergo DNAm analysis. In this study, we analyzed the DNAm data of these 2474 TWB participants. Written informed consent and blood and urine samples were provided by each individual when participating in the study. The TWB collected lifestyle factors such as smoking status through a face-to-face interview with health care professionals. Serum glucose and HbA_1c_ were measured with a Hitachi LST008 analyzer (Hitachi High-Technologies, Tokyo, Japan) and a Trinity Biotech Premier Hb9210 analyzer (Bray, Ireland/Kansas City, MO) after a fast of at least 6 h. FEV1 and FCV were measured through pulmonary function test machines. FVC is the amount of air forcibly exhaled from the lungs after taking the deepest breath, and FEV1 is the volume of air exhaled in the first second [46].

### Calculations of EAA (M)

The TWB quantified the blood DNAm levels of the 2474 participants through the Illumina Infinium MethylationEPIC BeadChip covering ~ 860,000 CpGs. The quality controls and normalization process for the DNAm data have been described elsewhere [35]. The DNAm Age Calculator from Horvath’s laboratory, https://dnamage.genetics.ucla.edu/new, was used to calculate four measures of EAA and the two components of GrimEAA: HannumEAA [26] (column “AgeAccelerationResidualHannum” of the DNAm Age Calculator output), IEAA [27] (column “IEAA”), PhenoEAA [28] (column “AgeAccelPheno”), GrimEAA [29, 30] (column “AgeAccelGrim2”), DNAm-based smoking pack-years [29, 30] (column “DNAmPACKYRS”), and DNAm PAI-1 levels [29, 30] (column “DNAmPAI1”). DunedinPACE [31] was obtained from the R package DunedinPACE (https://github.com/danbelsky/DunedinPACE).

### Statistical analysis

We performed 120 mediation analyses to investigate which of the six epigenetic measures (IEAA not included according to Table 2) plays the best role as a mediator between the five smoking variables and four health outcomes (6 × 5 × 4 = 120). Two right-skewed health outcomes, FG and HbA_1c_, were natural-log transformed to meet the normality assumption for linear models. Furthermore, all four health outcomes were standardized as *z*-scores. To be considered a plausible mediator, two requirements should be met: (1) the association between smoking (X) and EAA (M); and (2) the association between EAA (M) and the health outcome (Y) while controlling for the smoking variable (X).

Former smoking status (Table 3 and Fig. 1) and current smoking status (Table 4 and Fig. 2) were investigated through the following two models (M model and Y model):

1 $$\mathrm{EAA }\left(M\right) = {\beta }_{0M}+{\beta }_{{X}_{1}M}FS+{\beta }_{{X}_{2}M}CS+{{\varvec{\beta}}}_{{\varvec{C}}{\varvec{M}}}^{\prime}\mathbf{Covariates}+{\varepsilon }_{M};$$

2 $$\mathrm{Health outcome }\left(Y\right) = {\beta }_{0Y}+{\beta }_{{X}_{1}Y}FS+{\beta }_{{X}_{2}Y}CS+{\beta }_{MY}EAA \left(M\right)+{{\varvec{\beta}}}_{{\varvec{C}}{\varvec{Y}}}^{\prime}\mathbf{Covariates}+{\varepsilon }_{Y},$$

where *FS* and *CS* are dummy-coded variables for former smokers and current smokers (with non-smokers treated as the reference group); $\mathbf{Covariates}$ included age, sex, BMI, drinking status, regular exercise status, educational attainment, and five cell-type proportions (B lymphocytes, CD4^+^ T cells, CD8^+^ T cells, monocytes, and natural killer cells). ${\varepsilon }_{M}$ and ${\varepsilon }_{Y}$ are random error terms of the M model and Y model, respectively. The direct effect of current smoking on the health outcome was calculated by $\widehat{{\beta }_{{X}_{2}Y}}$, and the mediation effect was estimated by $\widehat{{\beta }_{{X}_{2}M}}\cdot \widehat{{\beta }_{MY}}$, where ^ represents the regression coefficients estimated from models (1) and (2). The total effect of current smoking on the health outcome was the sum of the direct and mediation effects, i.e., $\widehat{{\beta }_{{X}_{2}Y}}+\widehat{{\beta }_{{X}_{2}M}}\cdot \widehat{{\beta }_{MY}}$. The effect of former smoking was calculated similarly.

Regarding the analysis for former smokers’ pack-years (Table 5 and Fig. 3) and current smokers’ pack-years (Table 6 and Fig. 4), we considered the following two models:

3 $$\mathrm{EAA }\left(M\right) = {\beta }_{0M}+{\beta }_{{X}_{1}M}FPY+{\beta }_{{X}_{2}M}CPY+{{\varvec{\beta}}}_{{\varvec{C}}{\varvec{M}}}^{\prime}\mathbf{Covariates}+{\varepsilon }_{M};$$

4 $$\mathrm{Health outcome }\left(Y\right) = {\beta }_{0Y}+{\beta }_{{X}_{1}Y}FPY+{\beta }_{{X}_{2}Y}CPY+{\beta }_{MY}EAA \left(M\right)+{{\varvec{\beta}}}_{{\varvec{C}}{\varvec{Y}}}^{\prime}\mathbf{Covariates}+{\varepsilon }_{Y},$$

where *FPY* and *CPY* are variables coding former smokers’ pack-years and current smokers’ pack-years (with non-smokers coded as 0 in these two variables); $\mathbf{Covariates}$ have been described for models (1)–(2).

For analysis of years since smoking cessation (Table 7 and Fig. 5), only former smokers were analyzed by the following two models:

5 $$\mathrm{EAA }\left(M\right) = {\beta }_{0M}+{\beta }_{XM}YSC+{{\varvec{\beta}}}_{{\varvec{C}}{\varvec{M}}}^{\prime}\mathbf{Covariates}+{\varepsilon }_{M};$$

6 $$\mathrm{Health outcome }\left(Y\right) = {\beta }_{0Y}+{\beta }_{XY}YSC+{\beta }_{MY}EAA \left(M\right)+{{\varvec{\beta}}}_{{\varvec{C}}{\varvec{Y}}}^{\prime}\mathbf{Covariates}+{\varepsilon }_{Y},$$

where *YSC* is former smokers’ years since smoking cessation; $\mathbf{Covariates}$ included former smokers’ pack-years and the covariates described for models (1)–(2).

All statistical analyses were conducted with R software (version 4.2.3) [47], where the mediation analyses were performed using the R package **mediation** [48, 49]. We used the nonparametric bootstrap for the variance estimation by assigning the “boot = TRUE” option in the “*mediate*” function. The number of simulations was set at 2000 by setting “sims = 2000”. After obtaining the 120 *p*-values for the testing hypothesis: *H*_*0*_: mediation effect is 0 versus *H*_*1*_: mediation effect is not 0, we performed the Benjamini–Hochberg FDR control [50]. Mediation effects with FDR < 0.05 were considered statistically significant.

We calculated variance inflation factor (VIF) values to check multicollinearity. A VIF value larger than 5 is usually considered a threat of multicollinearity [51]. No multicollinearity among the explanatory variables was detected in models (1)–(6) or models in Table 2, for the analyses of 2474 individuals (largest VIF = 2.51) and 1243 male participants (largest VIF = 2.26).

## Supplementary Information

**Additional file 1:** **Table S1.** Basic characteristics of the 173,807 Taiwan Biobank (TWB) participants stratified by smoking status.** Table S2.** (Based on 173,807 Taiwan Biobank participants) Results of regressing health outcomes on smoking variables while adjusting for six covariates (chronological age (in years), sex (male vs. female), BMI (in kg/m2), drinking status (yes vs. no), performing regular exercise (yes vs. no), and educational attainment (an integer ranging from 1 to 7)).** Table S3.** (Only in males) Basic characteristics of the 1243 male participants stratified by smoking status.** Table S4.** (Only in males) Results of regressing each DNAm marker on smoking variables (based on models 1, 3, and 5).** Table S5.** (Only in males) Results of six DNAm markers in mediating the associations between former smoking status and four health outcomes.** Table S6.** (Only in males) Results of six DNAm markers in mediating the associations between current smoking status and four health outcomes.** Table S7.** (Only in males) Results of six DNAm markers in mediating the associations between former smokers’ pack-years and four health outcomes.** Table S8.** (Only in males) Results of six DNAm markers in mediating the associations between current smokers’ pack-years and four health outcomes.** Table S9.** (Only in males) Results of six DNAm markers in mediating the associations between years since smoking cessation and four health outcomes.** Figure S1.** Boxplots of the seven DNA methylation (DNAm) markers.** Figure S2.** (Both males and females) Heatmap of Pearson’s correlation coefficients among variables. The numbers in cells are Pearson’s correlation coefficients between pairwise variables.** Figure S3.** (Only in males) Heatmap of Pearson’s correlation coefficients among variables. The numbers in cells are Pearson’s correlation coefficients between pairwise variables.** Figure S4.** (Only in males) 95% confidence intervals of the effects of six DNAm markers in mediating the associations between former smoking status and four health outcomes.** Figure S5.** (Only in males) 95% confidence intervals of the effects of six DNAm markers in mediating the associations between current smoking status and four health outcomes.** Figure S6.** (Only in males) 95% confidence intervals of the effects of six DNAm markers in mediating the associations between former smokers’ pack-years and four health outcomes.** Figure S7.** (Only in males) 95% confidence intervals of the effects of six DNAm markers in mediating the associations between current smokers’ pack-years and four health outcomes.** Figure S8.** (Only in males) 95% confidence intervals of the effects of six DNAm markers in mediating the associations between years since smoking cessation and four health outcomes.** Figure S9.** (Both males and females) The original p-values (without FDR adjustment) of the mediation effects.** Figure S10.** (Only in males) The original p-values (without FDR adjustment) of the mediation effects.

## Data Availability

The datasets used and analyzed during the current study are available from https://www.twbiobank.org.tw/.

## Abbreviations

COPD: Chronic obstructive pulmonary disease
CpGs: Cytosine-phosphate-guanine sites
DNAm: DNA methylation
EAA: Epigenetic age acceleration
EWAS: Epigenome-wide association studies
FDR: False discovery rate
FEV1: Forced expiratory volume in 1.0 s
FG: Fasting glucose
FVC: Forced vital capacity
HbA_1c_: Hemoglobin A1C
HRS: Health and Retirement Study
IEAA: Intrinsic epigenetic age acceleration
PAI-1: Plasminogen activation inhibitor 1
SD: Standard deviation
TWB: Taiwan Biobank
VIF: Variance inflation factor
