# Supplementary material for: Epigenetic age acceleration mediates the association between smoking and diabetes-related outcomes
Source: Clin Epigenetics. 2023 Jun 3;15:94. doi: 10.1186/s13148-023-01512-x (PMC10239178; doi:10.1186/s13148-023-01512-x)
Supplement: Supplementary file 1 — Additional file 1: Table S1. Basic characteristics of the 173,807 Taiwan Biobank (TWB) participants stratified by smoking status. Table S2. (Based on 173,807 Taiwan Biobank participants) Results of regressing health outcomes on smoking variables while adjusting for six covariates (chronological age (in years), sex (male vs. female), BMI (in kg/m2), drinking status (yes vs. no), performing regular exercise (yes vs. no), and educational attainment (an integer ranging from 1 to 7)). Table S3. (Only in males) Basic characteristics of the 1243 male participants stratified by smoking status. Table S4. (Only in males) Results of regressing each DNAm marker on smoking variables (based on models 1, 3, and 5). Table S5. (Only in males) Results of six DNAm markers in mediating the associations between former smoking status and four health outcomes. Table S6. (Only in males) Results of six DNAm markers in mediating the associations between current smoking status and four health outcomes. Table S7. (Only in males) Results of six DNAm markers in mediating the associations between former smokers’ pack-years and four health outcomes. Table S8. (Only in males) Results of six DNAm markers in mediating the associations between current smokers’ pack-years and four health outcomes. Table S9. (Only in males) Results of six DNAm markers in mediating the associations between years since smoking cessation and four health outcomes. Figure S1. Boxplots of the seven DNA methylation (DNAm) markers. Figure S2. (Both males and females) Heatmap of Pearson’s correlation coefficients among variables. The numbers in cells are Pearson’s correlation coefficients between pairwise variables. Figure S3. (Only in males) Heatmap of Pearson’s correlation coefficients among variables. The numbers in cells are Pearson’s correlation coefficients between pairwise variables. Figure S4. (Only in males) 95% confidence intervals of the effects of six DNAm markers in mediating the associations between former smoking [file 13148_2023_1512_MOESM1_ESM.pdf]

Supplementary Materials

Epigenetic age acceleration mediates the association between smoking and diabetes-related outcomes

Xue-Yong Chang [1], Wan-Yu Lin [1,2,3]*

[1] Institute of Epidemiology and Preventive Medicine, College of Public Health, National Taiwan University, Taipei, Taiwan

[2] Master of Public Health Degree Program, College of Public Health, National Taiwan University, Taipei, Taiwan

[3] Department of Public Health, College of Public Health, National Taiwan University, Taipei, Taiwan

Running title: Epigenetic age acceleration mediates the associations between smoking and health outcomes.

* Corresponding author: Wan-Yu Lin, Ph.D.

Wan-Yu Lin, Ph.D. (http://orcid.org/0000-0002-3385-4702)

Room 501, No. 17, Xu-Zhou Road, Taipei 100, Taiwan

Phone/Fax: +886-2-33668106; E-mail: linwy@ntu.edu.tw

Supplemental Tables

TABLE S1 Basic characteristics of the 173,807 Taiwan Biobank (TWB) participants stratified by smoking status

TABLE S2 (Based on 173,807 Taiwan Biobank participants) Results of regressing health outcomes on smoking variables while adjusting for six covariates (chronological age (in years), sex (male vs. female), BMI (in kg/m^2), drinking status (yes vs. no), performing regular exercise (yes vs. no), and educational attainment (an integer ranging from 1 to 7))

TABLE S3 (Only in males) Basic characteristics of the 1,243 male participants stratified by smoking status

TABLE S4 (Only in males) Results of regressing each DNAm marker on smoking variables (based on models 1, 3, and 5)

TABLE S5 (Only in males) Results of six DNAm markers in mediating the associations between **former smoking status** and four health outcomes

TABLE S6 (Only in males) Results of six DNAm markers in mediating the associations between **current smoking status** and four health outcomes

TABLE S7 (Only in males) Results of six DNAm markers in mediating the associations between **former smokers' pack-years** and four health outcomes

TABLE S8 (Only in males) Results of six DNAm markers in mediating the associations between **current smokers' pack-years** and four health outcomes

TABLE S9 (Only in males) Results of six DNAm markers in mediating the associations between **years since smoking cessation** and four health outcomes

Supplemental Figures

FIGURE S1 Boxplots of the seven DNA methylation (DNAm) markers

FIGURE S2 (Both males and females) Heatmap of Pearson's correlation coefficients among variables. The numbers in cells are Pearson's correlation coefficients between pairwise variables.

FIGURE S3 (Only in males) Heatmap of Pearson's correlation coefficients among variables. The numbers in cells are Pearson's correlation coefficients between pairwise variables.

FIGURE S4 (Only in males) 95% confidence intervals of the effects of six DNAm markers in mediating the associations between **former smoking status** and four health outcomes.

FIGURE S5 (Only in males) 95% confidence intervals of the effects of six DNAm markers in mediating the associations between **current smoking status** and four health outcomes.

FIGURE S6 (Only in males) 95% confidence intervals of the effects of six DNAm markers in mediating the associations between **former smokers' pack-years** and four health outcomes.

FIGURE S7 (Only in males) 95% confidence intervals of the effects of six DNAm markers in mediating the associations between **current smokers' pack-years** and four health outcomes.

FIGURE S8 (Only in males) 95% confidence intervals of the effects of six DNAm markers in mediating the associations between **years since smoking cessation** and four health outcomes.

FIGURE S9 (Both males and females) The original *p*-values (without FDR adjustment) of the mediation effects

FIGURE S10 (Only in males) The original *p*-values (without FDR adjustment) of the mediation effects

Tables

TABLE S1 Basic characteristics of the 173,807 Taiwan Biobank (TWB) participants stratified by smoking status

	Non-smokers	Former smokers [a]	Current smokers [b]	*p*-value [c]
**Total**	140,450 (80.81%)	17,006 (9.78%)	16,351 (9.41%)	
**Age (year)**	48.5 (12.3)	52.5 (11.0)	46.2 (11.0)	< 2.2E-16
**Age range**	20 - 92	20 - 92	20 - 82	
**Sex (male)**	36642 (26.1%)	14017 (82.4%)	12786 (78.2%)	< 2.2E-16
**Drinking [d]**	4215 (3.0%)	2669 (15.7%)	3920 (24.0%)	< 2.2E-16
**Former smokers' pack-years**	---	10.7 (13.9)	---	
**Current smokers' pack-years**	---	---	18.2 (17.1)	
**Years since smoking cessation**	---	13.9 (10.5)	---	
**Regular exercise [e]**	54422 (38.7%)	8058 (47.4%)	4437 (27.1%)	1.9E-318
**Educational attainment [f]**	5.6 (1.0)	5.5 (0.9)	5.4 (0.9)	2.3E-230
**BMI (kg/m^2)**	24.0 (3.9)	25.4 (3.6)	25.3 (4.2)	< 2.2E-16
**FG (mg/dL)**	94.9 (18.9)	100.4 (24.3)	99.0 (26.2)	< 2.2E-16
**HbA_{1c} (%)**	5.7 (0.7)	5.9 (0.9)	5.8 (1.0)	4.5E-196
**FEV1 (L) [g]**	2.1 (0.8)	2.6 (0.8)	2.6 (0.9)	< 2.2E-16
**FVC (L) [g]**	2.6 (0.9)	3.2 (0.9)	3.3 (0.8)	< 2.2E-16

The characteristics are presented in *n* (%) or mean ± standard deviation.

[a] Former smokers were defined as those who “had previously smoked cigarettes for at least six months but had quit smoking for at least six months when participating in TWB.”

[b] Current smokers were defined as those who “had smoked cigarettes for at least six months and had not quit smoking when participating in TWB.”

[c] The *p*-value of testing the mean or proportion difference among non-smokers, former smokers, and current smokers was based on a Kruskal-Wallis test for continuous variables or a Chi-square test for sex, drinking, and regular exercise.

[d] Drinking was defined as those who “had a weekly intake of more than 150 mL of alcoholic beverages for at least six months and had not stopped drinking when participating in TWB”.

[e] Regular exercise was defined as “performing exercise for 30 minutes thrice a week”. ‘Exercise’ included leisure-time activities such as jogging, swimming, cycling, yoga, resistance training, hiking, etc.

[f] Educational attainment was an integer ranging from 1 to 7: 1 “illiterate”, 2 “no formal education but literate”, 3 “primary school graduate”, 4 “junior high school graduate”, 5 “senior high school graduate”, 6 “college graduate”, and 7 “Master’s or higher degree”.

[g] Only 71.7% of the 173,807 TWB participants underwent lung function examinations. The measurements of FEV1 and FVC were based on 124,665 individuals (100,467 non-smokers, 12,571 former smokers, and 11,627 current smokers).

		Former smoking status (yes vs. no)	Current smoking status (yes vs. no)	Former smokers' pack-years	Current smokers' pack-years	Years since smoking cessation
Health outcome	Regression model	Former and current smoking status are dummy-coded variables in the same regression model, as described in model (1). However, the response variable is health outcome here. Moreover, no cell-type proportions can be adjusted in the ~170,000 participants.		Former and current smokers' pack-years are variables in the same regression model, as described in model (3). However, the response variable is health outcome here. Moreover, no cell-type proportions can be adjusted in the ~170,000 participants.		Only former smokers were analyzed in this regression model, with former smokers' pack-years as an additional covariate, as described in model (5). However, the response variable is health outcome here. Moreover, no cell-type proportions can be adjusted in the ~170,000 participants.
FG	Effect (S.E.)	**0.035 (0.008)**	**0.06 (0.008)**	**0.003 (4.4E-04)**	**0.004 (3.2E-04)**	**-0.003 (0.001)**
	p-value	**2.20E-05**	**9.80E-13**	**3.50E-15**	**3.10E-32**	**0.0036**
HBA_{1C}	Effect (S.E.)	**0.022 (0.008)**	**0.098 (0.008)**	**0.004 (4.4E-04)**	**0.006 (3.2E-04)**	**-0.003 (0.001)**
	p-value	**0.0062**	**4.00E-32**	**2.90E-16**	**1.50E-77**	**0.0006**
FEV1	Effect (S.E.)	**0.017 (0.008)**	**-0.058 (0.008)**	**-0.003 (4.3E-04)**	**-0.005 (3.1E-04)**	**0.005 (0.001)**
	p-value	**0.0327**	**9.10E-12**	**5.20E-09**	**7.10E-64**	**9.40E-07**
FVC	Effect (S.E.)	**0.039 (0.007)**	-0.002 (0.008)	**-0.001 (4.0E-04)**	**-0.003 (2.9E-04)**	-2.0E-05 (0.001)
	p-value	**1.20E-07**	0.8208	**0.0189**	**3.70E-26**	0.9821

TABLE S2 (Based on 173,807 Taiwan Biobank participants) Results of regressing health outcomes on smoking variables while adjusting for six covariates (chronological age (in years), sex (male vs. female), BMI (in kg/m^2), drinking status (yes vs. no), performing regular exercise (yes vs. no), and educational attainment (an integer ranging from 1 to 7))

Significant results with $p < 0.05$ are highlighted in bold font. FG and HbA_{1c} were natural-log transformed and then standardized as *z*-scores; FEV1 and FVC were standardized as *z*-scores.

TABLE S3 (Only in males) Basic characteristics of the 1,243 male participants stratified by smoking status

	Non-smokers	Former smokers [a]	Current smokers [b]	*p*-value [c]
**Total**	736 (59.2%)	272 (21.9%)	235 (18.9%)	
**Age (year)**	49.2 (11.8)	53.7 (10.0)	49.5 (10.5)	1.1E-07
**Age range**	30 - 70	30 - 70	30 - 70	
**Drinking [d]**	55 (7.5%)	30 (11.0%)	62 (26.4%)	4.9E-14
**The number of pack-years for former smokers**		14.1 (14.5)		
**The number of pack-years for current smokers**			22.6 (20.8)	
**The time since smoking cessation**		12.9 (10.1)		
**Regular exercise [e]**	346 (47.0%)	167 (61.4%)	82 (34.9%)	1.1E-08
**Educational attainment [f]**	5.9 (0.8)	5.7 (0.9)	5.5 (0.8)	1.2E-10
**BMI (kg/m^2)**	25.0 (3.5)	25.5 (3.1)	25.7 (3.5)	0.0035
**FG (mg/dL)**	97.3 (17.5)	98.7 (14.7)	104.0 (36.6)	0.0010
**HbA_{1c} (%)**	5.7 (0.7)	5.8 (0.7)	5.9 (1.1)	0.0018
**FEV1 (L) [g]**	2.7 (0.8)	2.5 (0.8)	2.6 (0.8)	0.0256
**FVC (L) [g]**	3.6 (0.7)	3.5 (0.7)	3.7 (0.7)	0.1207

The characteristics are presented in *n* (%) or mean ± standard deviation.

[a] Former smokers were defined as those who “had previously smoked cigarettes for at least six months but had quit smoking for at least six months when participating in TWB.”

[b] Current smokers were defined as those who “had smoked cigarettes for at least six months and had not quit smoking when participating in TWB.”

[c] The *p*-value of testing the mean or proportion difference among non-smokers, former smokers, and current smokers was based on a Kruskal-Wallis test for continuous variables or a Chi-square test for sex, drinking, and regular exercise.

[d] Drinking was defined as those who “had a weekly intake of more than 150 mL of alcoholic beverages for at least six months and had not stopped drinking when participating in TWB”.

[e] Regular exercise was defined as “performing exercise for 30 minutes thrice a week”. ‘Exercise’ included leisure-time activities such as jogging, swimming, cycling, yoga, resistance training, hiking, etc.

[f] Educational attainment was an integer ranging from 1 to 7: 1 “illiterate”, 2 “no formal education but literate”, 3 “primary school graduate”, 4 “junior high school graduate”, 5 “senior high school graduate”, 6 “college graduate”, and 7 “Master’s or higher degree”.

[g] Only 65.2% of the 1,243 TWB male participants underwent lung function examinations. The measurements of FEV1 and FVC were based on 811 males (476 male non-smokers, 176 former male smokers, and 159 current male smokers).

	HannumEAA (in years)		IEAA (in years)		PhenoEAA (in years)		GrimEAA (in years)		DNAm-based smoking pack-years		DNAm PAI-1 (in pg/mL)		DunedinPACE	
	Effect (S.E.)	*p*-value	Effect (S.E.)	*p*-value	Effect (S.E.)	*p*-value	Effect (S.E.)	*p*-value	Effect (S.E.)	*p*-value	Effect (S.E.)	*p*-value	Effect (S.E.)	*p*-value
Former smoking status (yes vs. no) (based on model 1)	0.311 (0.24)	0.1955	0.118 (0.272)	0.6647	0.973 (0.31)	0.0017	**1.470 (0.223)**	**6.2E-11**	**5.153 (0.442)**	**9.1E-30**	216.032 (146.98)	0.1419	0.015 (0.007)	0.0201
Current smoking status (yes vs. no) (based on model 1)	**1.060 (0.266)**	**0.0001**	0.382 (0.3)	0.2044	**1.721 (0.344)**	**6.3E-07**	**5.83 (0.248)**	**2.2E-101**	**15.345 (0.529)**	**5.2E-140**	**988.208 (162.668)**	**1.7E-09**	**0.083 (0.007)**	**8.0E-29**
Former smokers' pack-years (based on model 3)	0.019 (0.011)	0.0878	0.019 (0.012)	0.1230	**0.047 (0.014)**	**0.0011**	**0.078 (0.011)**	**3.3E-13**	**0.244 (0.022)**	**6.8E-27**	11.951 (6.765)	0.0776	0.001 (3.1E-04)	0.0169
Current smokers' pack-years (based on model 3)	0.021 (0.008)	0.0077	0.002 (0.009)	0.8385	0.032 (0.01)	0.0023	**0.16 (0.008)**	**3.8E-79**	**0.528 (0.023)**	**4.4E-96**	**26.699 (4.891)**	**5.8E-08**	**0.002 (2.2E-04)**	**9.9E-21**
Years since smoking cessation (based on model 5)	-0.022 (0.026)	0.4030	-0.028 (0.029)	0.3238	-0.074 (0.032)	0.0212	**-0.105 (0.02)**	**3.7E-07**	**-0.343 (0.044)**	**2.4E-13**	-7.797 (13.52)	0.5646	-0.001 (0.001)	0.4151

TABLE S4 (Only in males) Results of regressing each DNAm marker on smoking variables (based on models 1, 3, and 5)

Significant results with $p < 0.05/(7x5) = 0.0014$ are highlighted in bold font (7: seven DNAm markers; 5: five smoking variables).

Outcome [a]	Total effect	95% confidence interval		*p*-value	Sample size
FG	-0.0033	-0.1485	0.1420	0.965	1241
HbA_{1c}	0.0344	-0.1131	0.1819	0.647	1241
FEV1	-0.0894	-0.2566	0.0778	0.294	811
FVC	0.0506	-0.0734	0.1747	0.423	811

Outcome	Mediator	Mediation effect [b]	95% confidence interval		FDR [c]	Proportion mediated [d]	Sample size
FG	HannumEAA	0.0034	-0.0030	0.0144	0.623	-103.0%	1237
HbA_{1c}		0.0063	-0.0036	0.0219	0.429	18.3%	1237
FEV1		0.0062	-0.0089	0.0238	0.623	-6.9%	807
FVC		0.0020	-0.0097	0.0155	0.813	4.0%	807
FG	PhenoEAA	0.0161	0.0016	0.0386	0.081	-487.9%	1240
HbA_{1c}		**0.0300**	**0.0093**	**0.0562**	**0.006**	87.2%	1240
FEV1		-0.0180	-0.0454	0.0067	0.305	20.1%	810
FVC		-0.0062	-0.0245	0.0104	0.669	-12.3%	810
FG	GrimEAA	**0.0753**	**0.0355**	**0.1231**	**<0.001**	-2281.8%	1238
HbA_{1c}		**0.0975**	**0.0562**	**0.1494**	**<0.001**	283.4%	1238
FEV1		-0.0102	-0.0416	0.0218	0.702	11.4%	809
FVC		-0.0152	-0.0394	0.0071	0.370	-30.0%	809
FG	DNAm-based pack-years	0.0596	0.0116	0.1135	0.060	-1806.1%	1191
HbA_{1c}		**0.0946**	**0.0460**	**0.1490**	**<0.001**	275.0%	1191
FEV1		0.0083	-0.0448	0.0658	0.897	-9.3%	777
FVC		0.0182	-0.0196	0.0581	0.623	36.0%	777
FG	DNAm PAI-1	0.0231	-0.0078	0.0559	0.314	-700.0%	1241
HbA_{1c}		0.0225	-0.0062	0.0534	0.299	65.4%	1241
FEV1		-0.0004	-0.0162	0.0159	0.961	0.4%	811
FVC		-0.0100	-0.0266	0.0004	0.175	-19.8%	811
FG	DunedinPACE	0.0163	0.0017	0.0376	0.079	-493.9%	1241
HbA_{1c}		0.0259	0.0049	0.0535	0.050	75.3%	1241
FEV1		0.0022	-0.0092	0.0162	0.804	-2.5%	811
FVC		-0.0035	-0.0150	0.0050	0.623	-6.9%	811

TABLE S5 (Only in males) Results of six DNAm markers in mediating the associations between **former smoking status** and four health outcomes

[a] Before mediation analysis, FG and HbA_{1c} were natural-log transformed and then standardized as *z*-scores; FEV1 and FVC were standardized as *z*-scores.

[b] Mediation effect is the effect of X (former smoking status) on Y (health outcome) through the mediator (DNAm marker).

[c] FDR (false discovery rate) represents the FDR-adjusted *p*-value. Significant effects with FDR < 0.05 are marked in bold.

[d] Proportion mediated was calculated by dividing the mediation effect by the total effect.

Outcome [a]	Total effect	95% confidence interval		*p*-value	Sample size
FG	**0.2846**	**0.1239**	**0.4453**	**0.001**	1241
HbA_{1c}	0.1361	-0.0272	0.2994	0.102	1241
FEV1	0.0443	-0.1390	0.2275	0.636	811
FVC	0.1331	-0.0029	0.2690	0.055	811

Outcome	Mediator	Mediation effect [b]	95% confidence interval		FDR [c]	Proportion mediated [d]	Sample size
FG	HannumEAA	0.0115	-0.0081	0.0330	0.414	4.0%	1237
HbA_{1c}		0.0214	0.0012	0.0469	0.117	15.7%	1237
FEV1		0.0098	-0.0144	0.0373	0.623	22.1%	807
FVC		0.0031	-0.0140	0.0230	0.804	2.3%	807
FG	PhenoEAA	0.0284	0.0039	0.0577	0.084	10.0%	1240
HbA_{1c}		**0.0530**	**0.0244**	**0.0892**	**<0.001**	38.9%	1240
FEV1		-0.0244	-0.0564	0.0077	0.293	-55.1%	810
FVC		-0.0084	-0.0318	0.0163	0.669	-6.3%	810
FG	GrimEAA	**0.2987**	**0.1600**	**0.4402**	**<0.001**	105.0%	1238
HbA_{1c}		**0.3869**	**0.2470**	**0.5487**	**<0.001**	284.3%	1238
FEV1		-0.0420	-0.1712	0.0852	0.669	-94.8%	809
FVC		-0.0626	-0.1499	0.0290	0.362	-47.0%	809
FG	DNAm-based pack-years	0.1774	0.0288	0.3302	0.084	62.3%	1191
HbA_{1c}		**0.2817**	**0.1345**	**0.4420**	**<0.001**	207.0%	1191
FEV1		0.0257	-0.1366	0.1935	0.868	58.0%	777
FVC		0.0566	-0.0682	0.1853	0.623	42.5%	777
FG	DNAm PAI-1	**0.1055**	**0.0586**	**0.1635**	**<0.001**	37.1%	1241
HbA_{1c}		**0.1027**	**0.0580**	**0.1552**	**<0.001**	75.5%	1241
FEV1		-0.0011	-0.0394	0.0361	0.958	-2.5%	811
FVC		-0.0257	-0.0548	0.0002	0.148	-19.3%	811
FG	DunedinPACE	**0.0884**	**0.0255**	**0.1604**	**0.017**	31.1%	1241
HbA_{1c}		**0.1406**	**0.0738**	**0.2196**	**<0.001**	103.3%	1241
FEV1		0.0117	-0.0435	0.0711	0.804	26.4%	811
FVC		-0.0191	-0.0624	0.0238	0.623	-14.4%	811

TABLE S6 (Only in males) Results of six DNAm markers in mediating the associations between **current smoking status** and four health outcomes

[a] Before mediation analysis, FG and HbA_{1c} were natural-log transformed and then standardized as *z*-scores; FEV1 and FVC were standardized as *z*-scores.

[b] Mediation effect is the effect of X (current smoking status) on Y (health outcome) through the mediator (DNAm marker).

[c] FDR (false discovery rate) represents the FDR-adjusted *p*-value. Significant effects with FDR < 0.05 are marked in bold.

[d] Proportion mediated was calculated by dividing the mediation effect by the total effect.

Outcome [a]	Total effect	95% confidence interval		*p*-value	Sample size
FG	0.0043	-0.0024	0.0109	0.207	1230
HbA_{1c}	0.0059	-0.0009	0.0126	0.090	1230
FEV1	-0.0011	-0.0089	0.0068	0.790	803
FVC	-0.0002	-0.0061	0.0056	0.939	803

Outcome	Mediator	Mediation effect [b]	95% confidence interval		FDR [c]	Proportion mediated [d]	Sample size
FG	HannumEAA	0.0002	-0.0002	0.0008	0.532	4.7%	1226
HbA_{1c}		0.0004	-0.0001	0.0011	0.278	6.8%	1226
FEV1		0.0004	-0.0005	0.0013	0.623	-36.4%	799
FVC		0.0002	-0.0005	0.0009	0.736	-100.0%	799
FG	PhenoEAA	0.0007	2.0E-05	0.0017	0.120	16.3%	1229
HbA_{1c}		**0.0014**	**0.0004**	**0.0027**	**<0.001**	23.7%	1229
FEV1		-0.0008	-0.0021	0.0002	0.314	72.7%	802
FVC		-0.0002	-0.0010	0.0007	0.804	100.0%	802
FG	GrimEAA	**0.0033**	**0.0015**	**0.0056**	**<0.001**	76.7%	1227
HbA_{1c}		**0.0045**	**0.0024**	**0.0069**	**<0.001**	76.3%	1227
FEV1		-0.0001	-0.0018	0.0016	0.958	9.1%	801
FVC		0.0001	-0.0012	0.0013	0.958	-50.0%	801
FG	DNAm-based pack-years	0.0018	-0.0006	0.0041	0.314	41.9%	1180
HbA_{1c}		0.0024	-1.0E-05	0.0049	0.148	40.7%	1180
FEV1		0.0010	-0.0016	0.0036	0.635	-90.9%	769
FVC		0.0022	0.0005	0.0042	0.060	-1100.0%	769
FG	DNAm PAI-1	0.0013	1.0E-05	0.0026	0.148	30.2%	1230
HbA_{1c}		0.0012	0.0001	0.0026	0.117	20.3%	1230
FEV1		4.0E-05	-0.0005	0.0007	0.942	-3.6%	803
FVC		-0.0003	-0.0008	0.0001	0.394	150.0%	803
FG	DunedinPACE	0.0007	0.0001	0.0017	0.073	16.3%	1230
HbA_{1c}		**0.0012**	**0.0003**	**0.0024**	**0.022**	20.3%	1230
FEV1		0.0002	-0.0004	0.0010	0.702	-18.2%	803
FVC		-0.0001	-0.0006	0.0005	0.942	50.0%	803

TABLE S7 (Only in males) Results of six DNAm markers in mediating the associations between **former smokers' pack-years** and four health outcomes

[a] Before mediation analysis, FG and HbA_{1c} were natural-log transformed and then standardized as *z*-scores; FEV1 and FVC were standardized as *z*-scores.

[b] Mediation effect is the effect of X (former smokers' pack-years) on Y (health outcome) through the mediator (DNAm marker).

[c] FDR (false discovery rate) represents the FDR-adjusted *p*-value. Significant effects with FDR < 0.05 are marked in bold.

[d] Proportion mediated was calculated by dividing the mediation effect by the total effect.

Outcome [a]	Total effect	95% confidence interval		*p*-value	Sample size
FG	**0.0119**	**0.0070**	**0.0167**	**1.5E-06**	1230
HbA_{1c}	**0.0061**	**0.0012**	**0.0110**	**0.015**	1230
FEV1	0.0003	-0.0051	0.0058	0.908	803
FVC	0.0001	-0.0039	0.0042	0.955	803

Outcome	Mediator	Mediation effect [b]	95% confidence interval		FDR [c]	Proportion mediated [d]	Sample size
FG	HannumEAA	0.0002	-0.0002	0.0008	0.464	1.7%	1226
HbA_{1c}		0.0004	0.0000	0.0010	0.148	6.6%	1226
FEV1		0.0002	-0.0003	0.0009	0.623	66.7%	799
FVC		0.0001	-0.0003	0.0005	0.766	100.0%	799
FG	PhenoEAA	0.0005	4.0E-05	0.0011	0.116	4.2%	1229
HbA_{1c}		**0.0009**	**0.0003**	**0.0018**	**<0.001**	14.8%	1229
FEV1		-0.0004	-0.0011	0.0001	0.314	-133.3%	802
FVC		-0.0001	-0.0005	0.0004	0.804	-100.0%	802
FG	GrimEAA	**0.0068**	**0.0029**	**0.0110**	**<0.001**	57.1%	1227
HbA_{1c}		**0.0091**	**0.0053**	**0.0136**	**<0.001**	149.2%	1227
FEV1		-0.0002	-0.0034	0.0030	0.958	-66.7%	801
FVC		0.0001	-0.0022	0.0025	0.958	100.0%	801
FG	DNAm-based pack-years	0.0038	-0.0017	0.0093	0.362	31.9%	1180
HbA_{1c}		0.0052	0.0003	0.0106	0.117	85.2%	1180
FEV1		0.0019	-0.0029	0.0080	0.669	633.3%	769
FVC		0.0044	0.0008	0.0091	0.080	4400.0%	769
FG	DNAm PAI-1	**0.0028**	**0.0014**	**0.0045**	**<0.001**	23.5%	1230
HbA_{1c}		**0.0028**	**0.0015**	**0.0045**	**<0.001**	45.9%	1230
FEV1		0.0001	-0.0008	0.0010	0.945	33.3%	803
FVC		-0.0004	-0.0012	0.0002	0.331	-400.0%	803
FG	DunedinPACE	0.0020	0.0004	0.0039	0.073	16.8%	1230
HbA_{1c}		**0.0034**	**0.0016**	**0.0055**	**<0.001**	55.7%	1230
FEV1		0.0004	-0.0010	0.0019	0.723	133.3%	803
FVC		-0.0001	-0.0011	0.0010	0.945	-100.0%	803

TABLE S8 (Only in males) Results of six DNAm markers in mediating the associations between **current smokers' pack-years** and four health outcomes

[a] Before mediation analysis, FG and HbA_{1c} were natural-log transformed and then standardized as *z*-scores; FEV1 and FVC were standardized as *z*-scores.

[b] Mediation effect is the effect of X (current smokers' pack-years) on Y (health outcome) through the mediator (DNAm marker).

[c] FDR (false discovery rate) represents the FDR-adjusted *p*-value. Significant effects with FDR < 0.05 are marked in bold.

[d] Proportion mediated was calculated by dividing the mediation effect by the total effect.

Outcome [a]	Total effect	95% confidence interval		*p*-value	Sample size
FG	-0.0008	-0.0133	0.0117	0.900	263
HbA_{1c}	-0.0034	-0.0167	0.0098	0.609	263
FEV1	-0.0047	-0.0212	0.0118	0.574	170
FVC	-0.0059	-0.0182	0.0064	0.347	170

Outcome	Mediator	Mediation effect [b]	95% confidence interval		FDR [c]	Proportion mediated [d]	Sample size
FG	HannumEAA	-0.0004	-0.0026	0.0009	0.766	50.0%	262
HbA_{1c}		-0.0006	-0.0031	0.0010	0.669	17.6%	262
FEV1		0.0001	-0.0031	0.0035	0.961	-2.1%	169
FVC		0.0012	-0.0003	0.0039	0.314	-20.3%	169
FG	PhenoEAA	-0.0014	-0.0046	0.0005	0.362	175.0%	263
HbA_{1c}		-0.0017	-0.0049	0.0003	0.293	50.0%	263
FEV1		0.0044	-0.0003	0.0105	0.175	-93.6%	170
FVC		0.0028	-0.0001	0.0063	0.158	-47.5%	170
FG	GrimEAA	-0.0018	-0.0063	0.0026	0.623	225.0%	263
HbA_{1c}		-0.0040	-0.0086	-0.0001	0.148	117.6%	263
FEV1		0.0028	-0.0025	0.0092	0.532	-59.6%	170
FVC		**0.0050**	**0.0014**	**0.0096**	**0.042**	-84.7%	170
FG	DNAm-based pack-years	-0.0022	-0.0091	0.0038	0.669	275.0%	261
HbA_{1c}		-0.0041	-0.0096	0.0011	0.280	120.6%	261
FEV1		0.0003	-0.0082	0.0087	0.972	-6.4%	169
FVC		-0.0020	-0.0081	0.0046	0.702	33.9%	169
FG	DNAm PAI-1	-0.0004	-0.0026	0.0010	0.734	50.0%	263
HbA_{1c}		-0.0007	-0.0038	0.0014	0.734	20.6%	263
FEV1		-0.0005	-0.0032	0.0012	0.734	10.6%	170
FVC		0.0005	-0.0009	0.0024	0.681	-8.5%	170
FG	DunedinPACE	-0.0001	-0.0014	0.0013	0.972	12.5%	263
HbA_{1c}		-0.0006	-0.0029	0.0012	0.674	17.6%	263
FEV1		0.0008	-0.0013	0.0037	0.669	-17.0%	170
FVC		0.0017	-0.0009	0.0052	0.405	-28.8%	170

TABLE S9 (Only in males) Results of six DNAm markers in mediating the associations between **years since smoking cessation** and four health outcomes

[a] Before mediation analysis, FG and HbA_{1c} were natural-log transformed and then standardized as *z*-scores; FEV1 and FVC were standardized as *z*-scores.

[b] Mediation effect is the effect of X (years since smoking cessation) on Y (health outcome) through the mediator (EAA).

[c] FDR (false discovery rate) represents the FDR-adjusted *p*-value. Significant effects with FDR < 0.05 are marked in bold.

[d] Proportion mediated was calculated by dividing the mediation effect by the total effect.

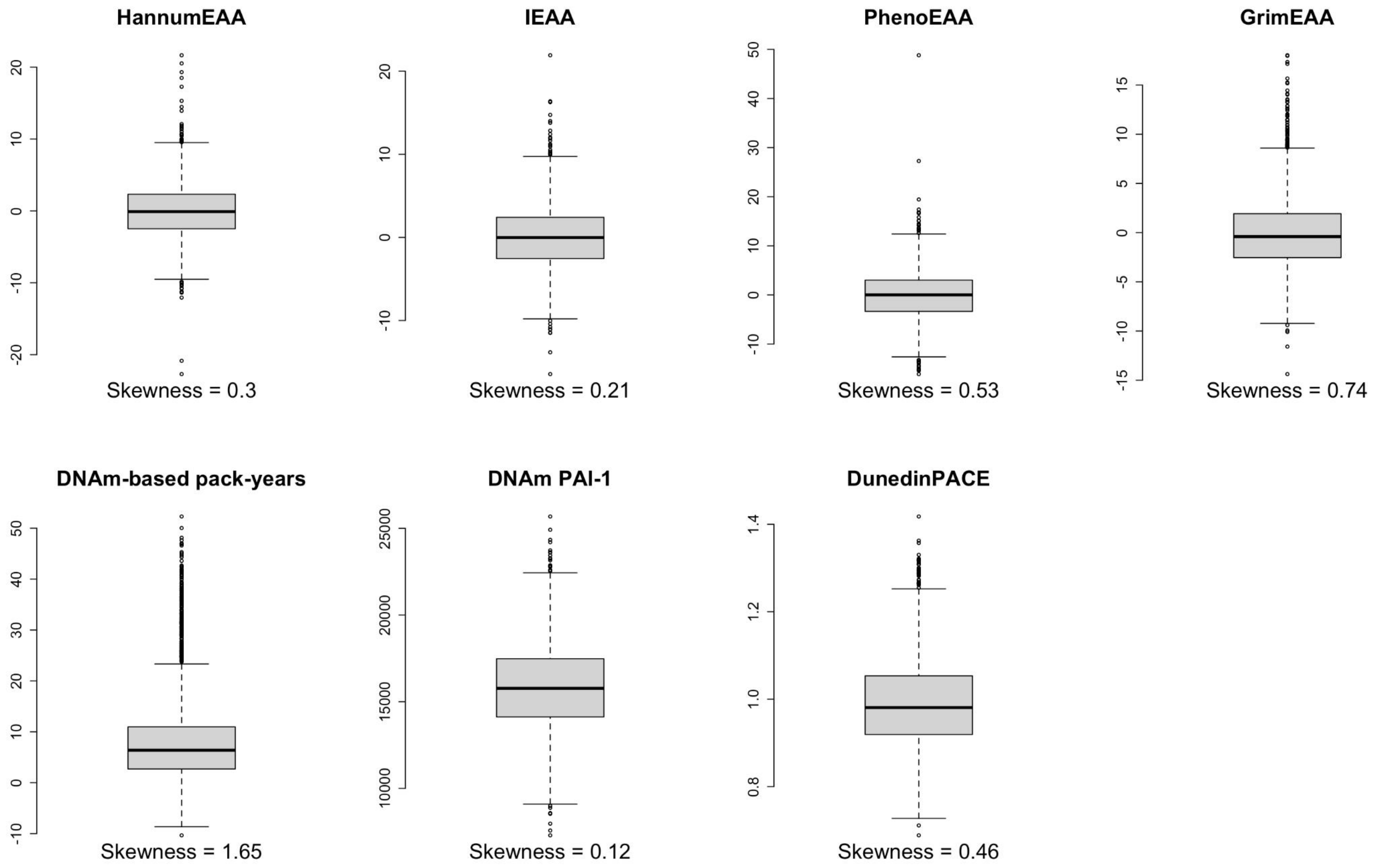

FIGURE S1 Boxplots of the seven DNA methylation (DNAm) markers

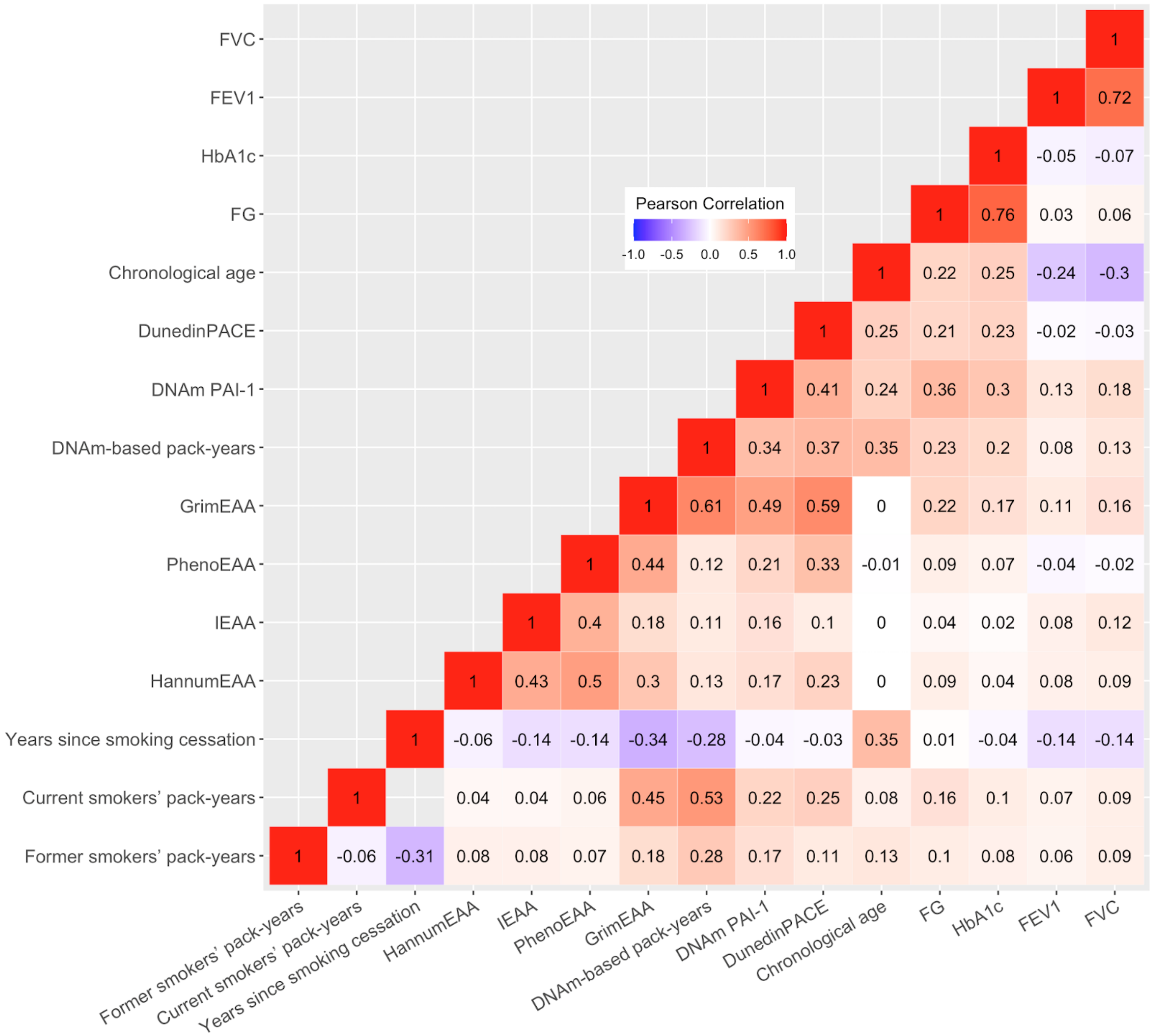

FIGURE S2 (Both males and females) Heatmap of Pearson's correlation coefficients among variables. The numbers in cells are Pearson's correlation coefficients between pairwise variables.

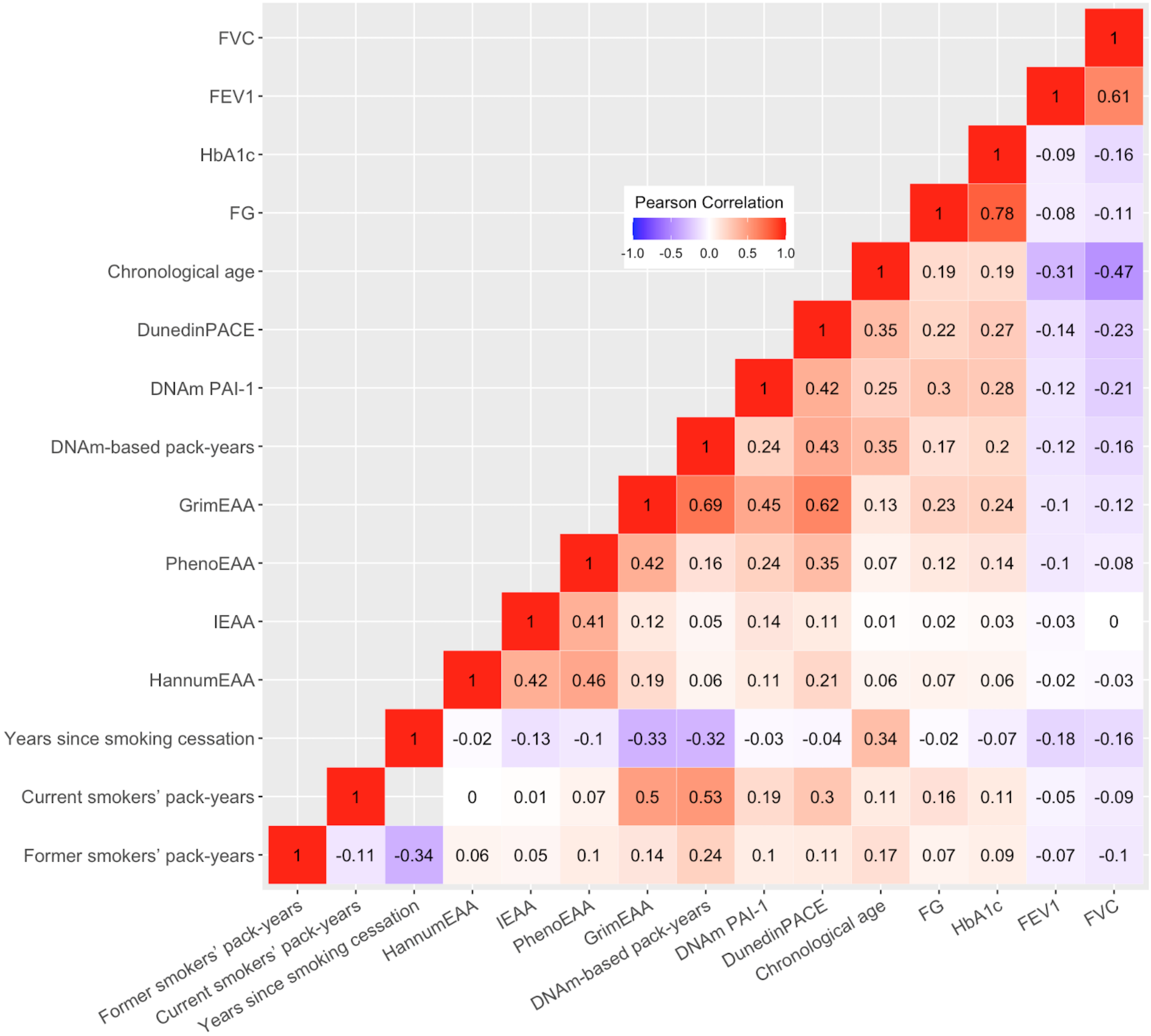

FIGURE S3 (Only in males) Heatmap of Pearson's correlation coefficients among variables. The numbers in cells are Pearson's correlation coefficients between pairwise variables.

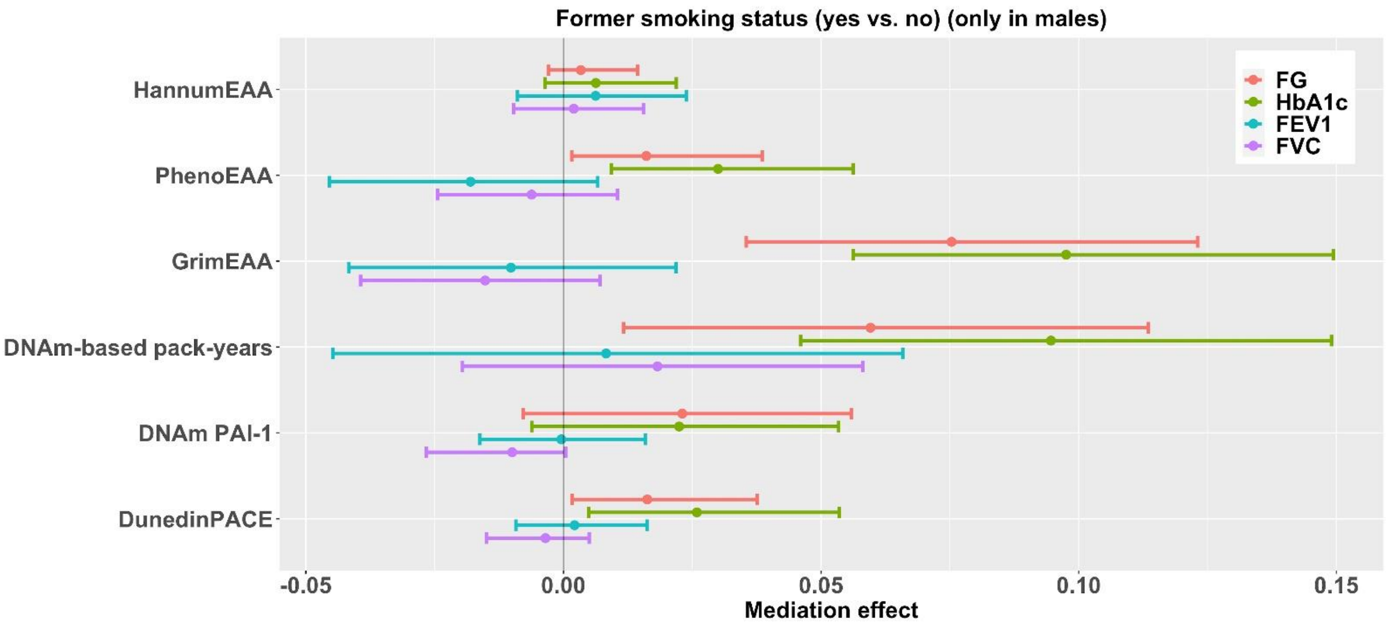

FIGURE S4 (Only in males) 95% confidence intervals of the effects of six DNAm markers in mediating the associations between **former smoking status** and four health outcomes. Before mediation analysis, FG and HbA_{1c} were natural-log transformed and then standardized as *z*-scores; FEV1 and FVC were standardized as *z*-scores.

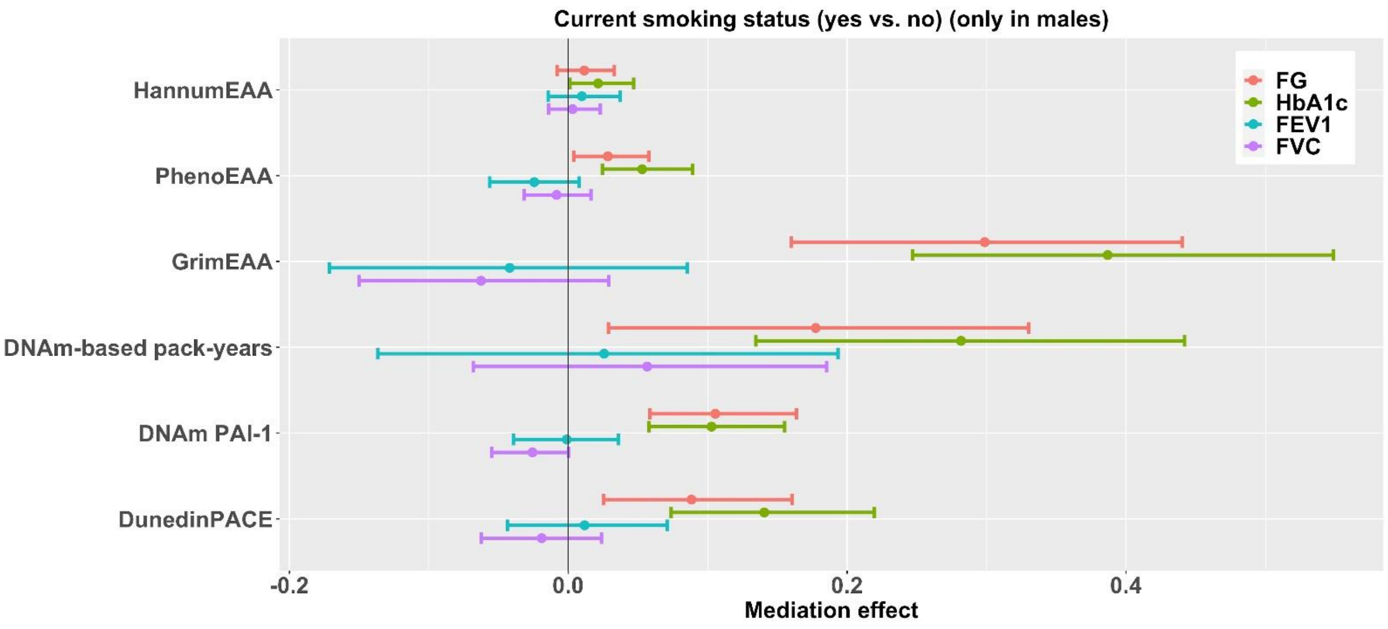

FIGURE S5 (Only in males) 95% confidence intervals of the effects of six DNAm markers in mediating the associations between **current smoking status** and four health outcomes. Before mediation analysis, FG and HbA_{1c} were natural-log transformed and then standardized as *z*-scores; FEV1 and FVC were standardized as *z*-scores.

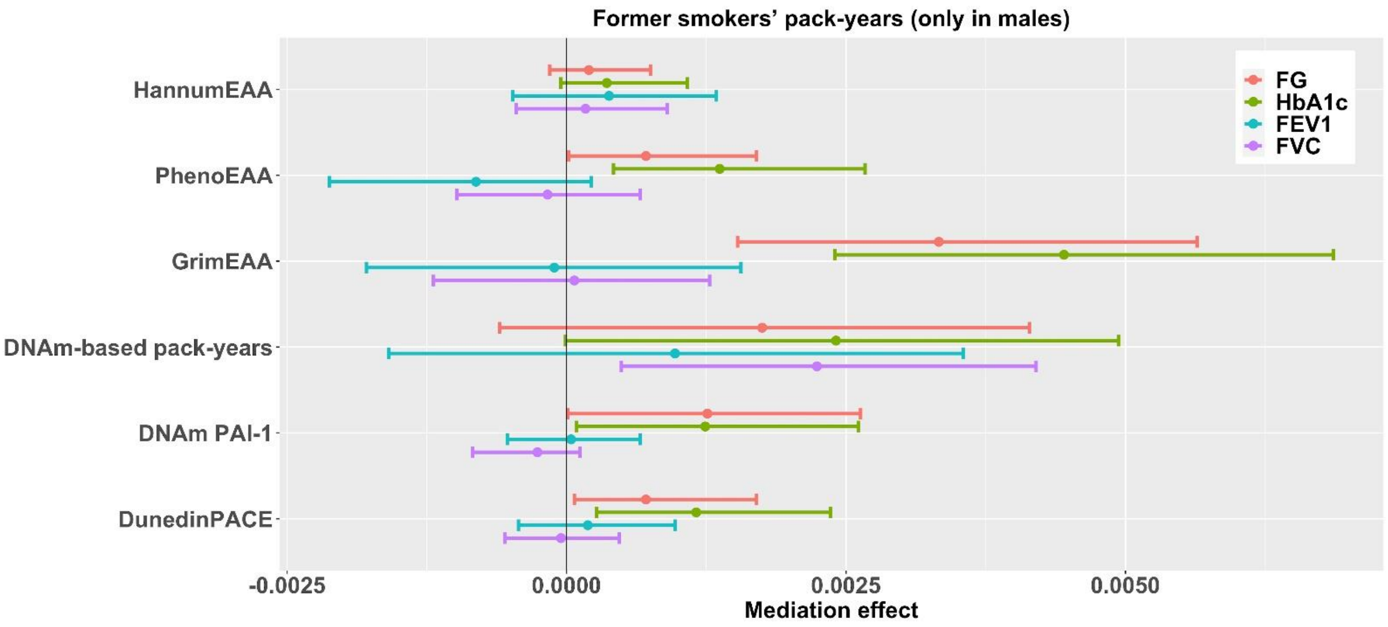

FIGURE S6 (Only in males) 95% confidence intervals of the effects of six DNAm markers in mediating the associations between **former smokers' pack-years** and four health outcomes. Before mediation analysis, FG and HbA_{1c} were natural-log transformed and then standardized as *z*-scores; FEV1 and FVC were standardized as *z*-scores.

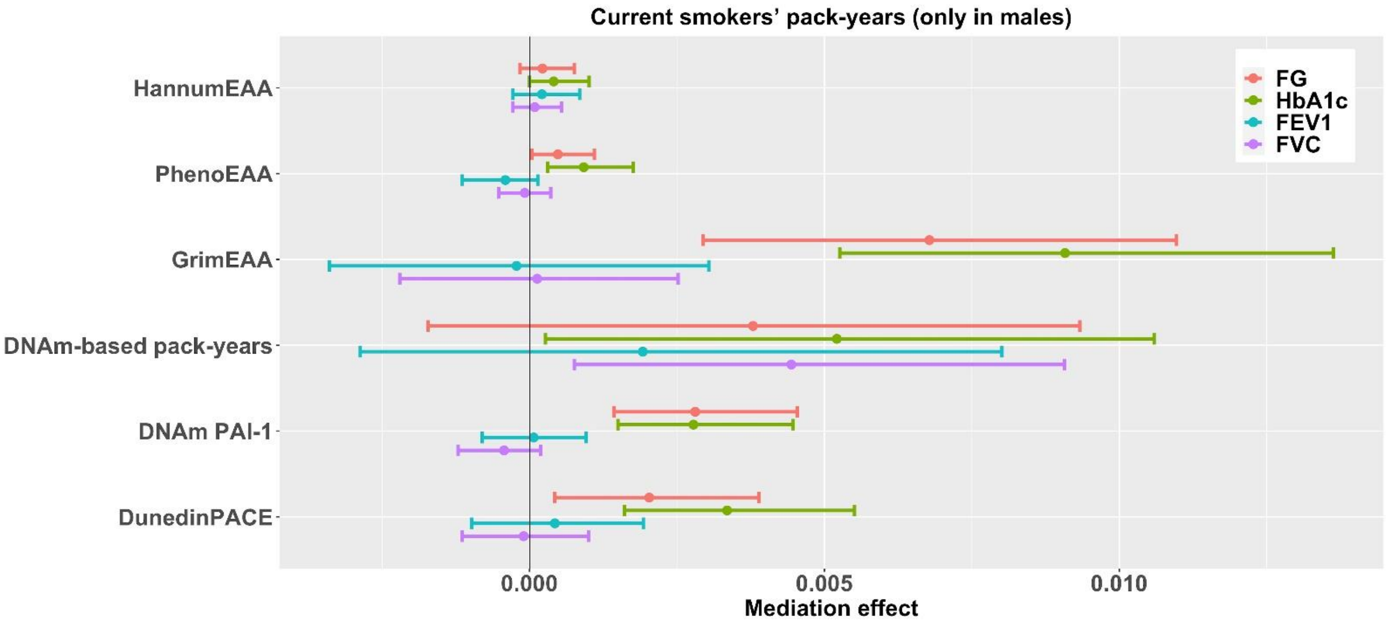

FIGURE S7 (Only in males) 95% confidence intervals of the effects of six DNAm markers in mediating the associations between **current smokers' pack-years** and four health outcomes. Before mediation analysis, FG and HbA_{1c} were natural-log transformed and then standardized as *z*-scores; FEV1 and FVC were standardized as *z*-scores.

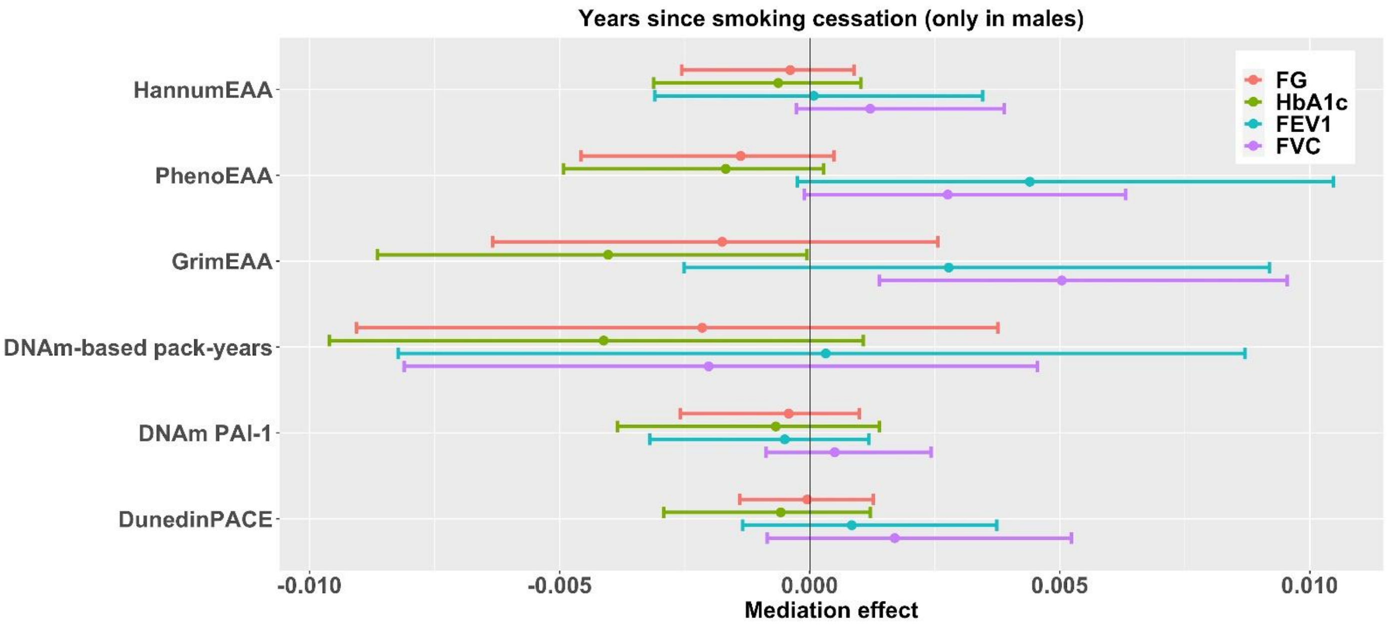

FIGURE S8 (Only in males) 95% confidence intervals of the effects of six DNAm markers in mediating the associations between **years since smoking cessation** and four health outcomes. Before mediation analysis, FG and HbA_{1c} were natural-log transformed and then standardized as *z*-scores; FEV1 and FVC were standardized as *z*-scores.

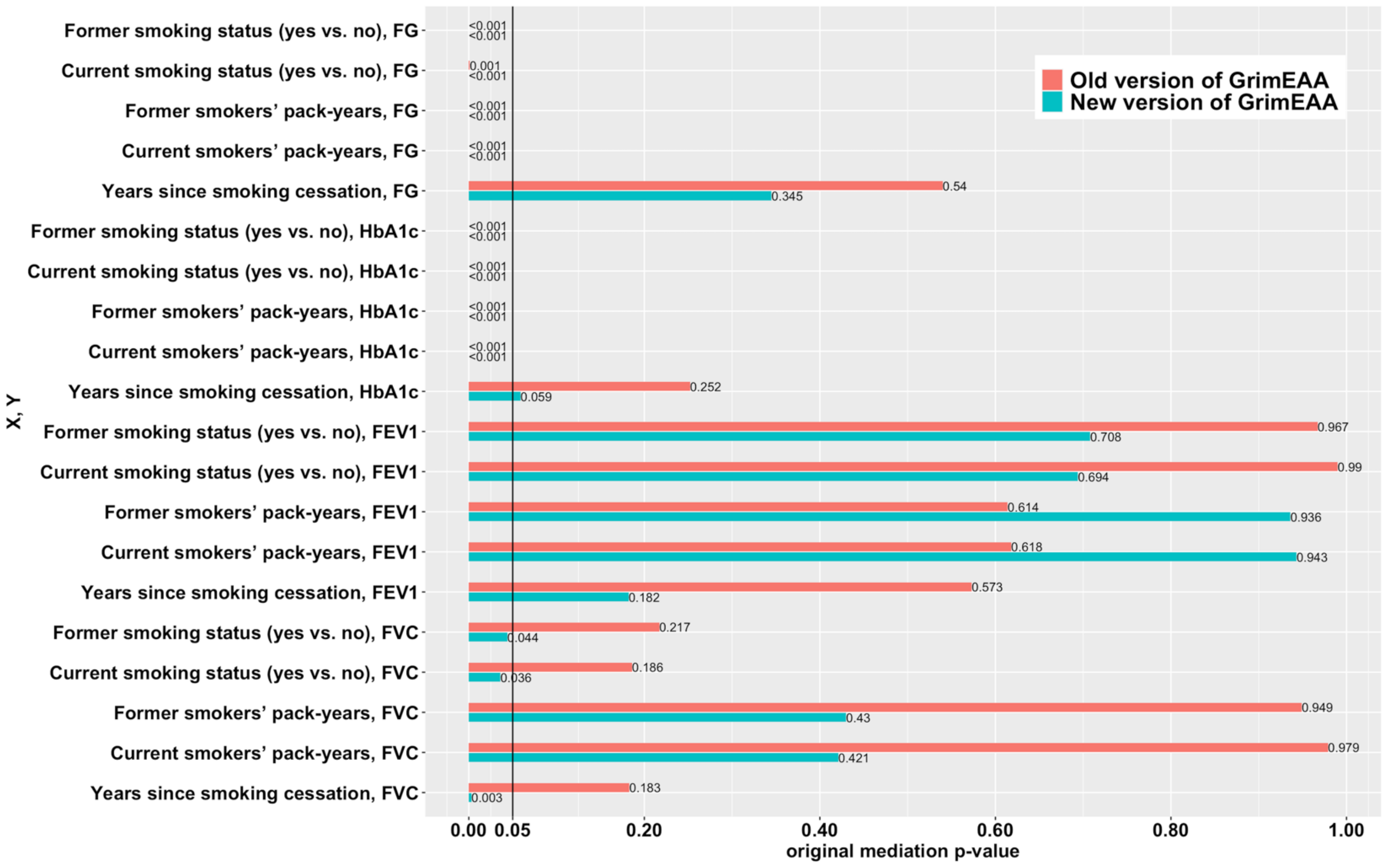

FIGURE S9 (Both males and females) The original *p*-values (without FDR adjustment) of the mediation effects

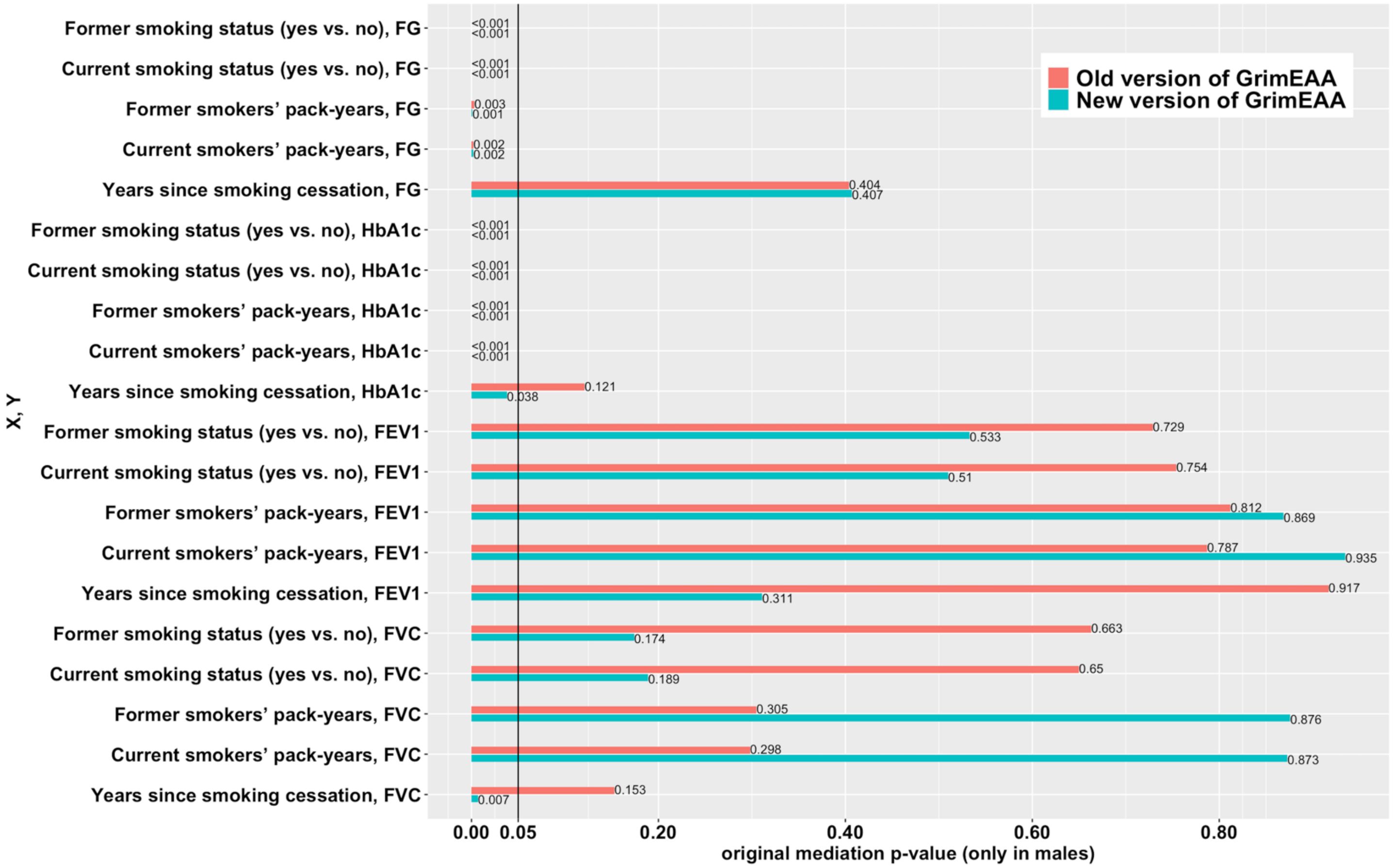

FIGURE S10 (Only in males) The original *p*-values (without FDR adjustment) of the mediation effects
